# Endogenous Nitric Oxide Can Enhance Oxidative Stress Caused by Air Pollutants and Explain Higher Susceptibility of Individuals with Inflammatory Disorders

**DOI:** 10.1021/acs.est.3c07010

**Published:** 2024-01-18

**Authors:** Steven Lelieveld, Jos Lelieveld, Ashmi Mishra, Andreas Daiber, Andrea Pozzer, Ulrich Pöschl, Thomas Berkemeier

**Affiliations:** † Multiphase Chemistry Department, Max Planck Institute for Chemistry, Mainz 55128, Germany; ‡ Atmospheric Chemistry Department, Max Planck Institute for Chemistry, Mainz 55128, Germany; § Climate and Atmosphere Research Center, the Cyprus Institute, Nicosia 2121, Cyprus; ∥ Department of Cardiology, University Medical Center of the Johannes Gutenberg University, Mainz 55131, Germany; ⊥ German Center for Cardiovascular Research (DZHK), Partner Site Rhine-Main, Mainz 55131, Germany

**Keywords:** air pollution, health effects, particulate matter, oxidative stress, inflammation

## Abstract

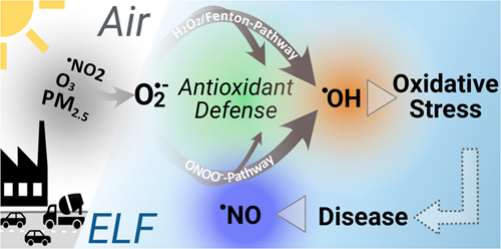

Air pollution causes morbidity and excess mortality. In the epithelial lining fluid of the respiratory tract, air pollutants trigger a chemical reaction sequence that causes the formation of noxious hydroxyl radicals that drive oxidative stress. For hitherto unknown reasons, individuals with pre-existing inflammatory disorders are particularly susceptible to air pollution. Through detailed multiphase chemical kinetic analysis, we show that the commonly elevated concentrations of endogenous nitric oxide in diseased individuals can increase the production of hydroxyl radicals via peroxynitrite formation. Our findings offer a molecular rationale of how adverse health effects and oxidative stress caused by air pollutants may be exacerbated by inflammatory disorders.

## Abstract 2

Elevated nitric oxide in individuals with inflammatory disorders enhances oxidative stress and ^•^OH production caused by air pollutants.

## Introduction

Air pollution is a leading cause of global morbidity and excess mortality.^1,2^ Pollutants include fine particulate matter with a diameter less than 2.5 μm (PM_2.5_) and gaseous oxidants such as ozone (O_3_) and nitrogen dioxide (^•^NO_2_).^3^ Exposure to air pollution may cause or exacerbate cardiovascular and respiratory diseases through excessive formation of reactive species such as hydroxyl radicals (^•^OH) in the epithelial lining fluid (ELF), inducing oxidative stress.^4−6^

Individuals predisposed with inflammatory disorders, such as asthma,^7^ chronic obstructive pulmonary disease (COPD),^8^ or cystic fibrosis,^9^ are especially susceptible to air pollution, but the underlying molecular mechanisms have been unclear.^10,11^ Elevated nitric oxide (^•^NO) concentrations from inducible nitric oxide synthase (iNOS) in the lungs have been observed in individuals with these inflammatory disorders.^12−15^ Exhaled ^•^NO (eNO) can reach volume mixing ratios up to several tens of ppb in the breath, exceeding typical ambient ^•^NO concentrations.^16−18^

^•^NO is a ubiquitous vaso- and bronchodilator involved in cardiovascular and respiratory regulation and plays a pivotal role in mediating apoptosis and inflammation.^19,20^ In the ELF, ^•^NO reacts with superoxide (O_2_^•–^) to form the potent oxidant and nitration agent peroxynitrite (ONOO^–^), which can act as an in situ source of ^•^OH.^21−25^ Increased production of ^•^OH due to ^•^NO has been observed in in vitro studies,^25−27^ but the effect of ^•^NO on air pollution-induced ^•^OH formation and oxidative stress has not been resolved. As O_2_^•–^ also acts as a source of ^•^OH in the presence of transition metals,^4,28^ it remains unclear whether scavenging of O_2_^•–^ and production of ONOO^–^ causes a net increase or net decrease of ^•^OH production in the ELF.

The kinetic multilayer model of surface and bulk chemistry in the ELF (KM-SUB-ELF) describes the multiphase chemical interactions at the air–body interface and has been used to quantify chemical dose–response relationships between ambient air pollution and reactive oxygen species (ROS) production in the ELF.^4,28,29^ Further, KM-SUB-ELF-predicted ROS concentrations have been used to connect long-term exposure to transition metals in PM_2.5_ with cardiovascular mortality.^30^ The model was also used to compare O_2_^•–^ production rates from macrophages with those from PM_2.5_ constituents in the ELF^31^ to investigate the ambient and endogenous sources of H_2_O_2_ in the lung^29^ and to estimate the concentration of nitrotyrosine, a biomarker for oxidative stress, in the ELF.^32^

In this study, we extended KM-SUB-ELF by endogenous ^•^NO and associated chemical reactions. The model offers insights into the production, interconversion, and scavenging of reactive species by pollutants, antioxidants, and antioxidant enzymes and thus allows quantitative apportionment of oxidative stress to individual constituents of air pollution and endogenous species. Furthermore, we use observational case-control studies with asthma, COPD, and cystic fibrosis patients to discuss our findings in the context of increased air pollution susceptibility in diseased individuals with elevated levels of ^•^NO.

## Materials and Methods

We extended and used the kinetic multilayer model of surface and bulk chemistry in the epithelial lining fluid (KM-SUB-ELF).^4,28,33^ The process-level flux model describes the multiphase chemical interactions between inhaled air pollutants and endogenous substances in the ELF with three distinct model compartments: the gas phase of the respiratory tract, the hydrophobic surfactant layer of the ELF, and the aqueous ELF. Species’ spatial and temporal evolution is calculated by solving a system of ordinary differential equations using MATLAB software. The model considers the airflow between ambient air and the respiratory tract (breathing), absorption and desorption of volatile species into and from the surfactant layer, exchange between the surfactant layer and aqueous ELF, diffusion within the aqueous ELF, and chemical reactions in all model compartments. In Table S1, 139 chemical reactions are listed with their respective rate coefficients. All reaction rate coefficients have been reported previously in the literature, and none were fitted in this study. In previous studies, KM-SUB-ELF has been fitted and validated using in vitro and in vivo observations.^4,28,29,32^ This includes chemical assays describing the ROS production of redox-active PM_2.5_ constituents,^34−37^ H_2_O_2_ depletion in lavage fluid of humans by catalase,^38^ and H_2_O_2_ and ^•^OH production of PM_2.5_ constituents in aqueous solutions mimicking ELF.^39,40^^32^

The ELF surfactant layer contains a monolayer of lipids (1-palmitoyl-2-oleoylglycerol) and includes proteins (surfactant protein B) and 200 μM α-tocopherol. The aqueous ELF is subdivided into five diffusion layers and contains antioxidants and antioxidant enzymes, i.e., glutathione, ascorbic acid, uric acid, superoxide dismutase (SOD), catalase, and glutathione peroxidase. The antioxidant and enzyme concentrations in the ELF are based on average concentrations adopted from studies using healthy volunteers,^38,41^ detailed in the supplementary text, and held constant to simulate fast replenishment. The pH of the ELF was assumed to be 7 and constant. Acids and conjugate bases are assumed to maintain equilibrium and are determined using the p*K*_a_ of the acid–base pairs. The simulated exposure time in this study is 2 h, which is spent with a constant lung ventilation rate of 1.5 m^3^ h^–1^. Dissolved particulate pollutant concentrations, i.e., iron (Fe), copper (Cu), secondary organic aerosol (SOA), and quinones, in the ELF are calculated based on their particulate mass fraction, deposition fraction, and solubility. For the negative ions of transition metals, Fe and Cu, we assume water-soluble fractions of 10 and 40%, respectively, while SOA and quinones are assumed to be entirely soluble. Note that transition metal solubilities can vary depending on the PM_2.5_ source and pH and thus constitute a source of uncertainty in this study. The fraction of PM_2.5_ that deposits in the ELF is assumed to be 45%. Because ^**•**^NO_2_ and PM_2.5_ are often coemitted and of similar mass concentration in the atmosphere, their ambient levels were assumed to be equal in all pollution scenarios investigated in this study. Ambient ^**•**^NO concentrations are typically lower than exhaled ^**•**^NO concentrations, and their effect are discussed in Section S2.

## Results and Discussion

**Figure 1:**
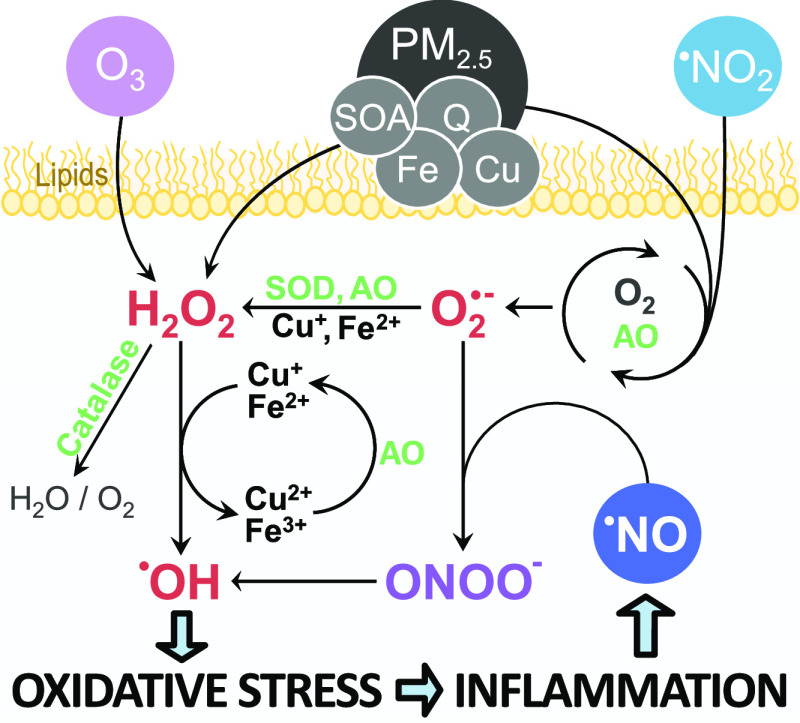
Production, interconversion, and scavenging of reactive oxygen species in the epithelial lining fluid (ELF). Reactions of PM_2.5_ constituents (copper, iron, quinones) and ^•^NO_2_ with antioxidants (AO) lead to superoxide (O_2_^•–^) formation. O_2_^•–^ is converted into hydrogen peroxide (H_2_O_2_) by superoxide dismutase (SOD) and AO, followed by scavenging of H_2_O_2_ through catalase. Secondary organic aerosol (SOA) components also produce H_2_O_2_ and hydroxyl radicals (^•^OH). Copper (Cu^+^) and iron (Fe^2+^) ions compete with catalase to form ^•^OH, the most reactive species driving oxidative stress. Through reactions of PM_2.5_ constituents with antioxidants, redox cycling is sustained in the ELF. Inflammation is promoted by oxidative stress and is associated with increased levels of nitric oxide (^•^NO), which competes with the antioxidant defense system for O_2_^•–^ to form peroxynitrite (ONOO^–^), a labile compound that can decompose to form ^•^OH.

Figure 1 illustrates the main reaction pathways of reactive oxygen species and reactive nitrogen species that result from air pollutant exposure to PM_2.5_, ^•^NO_2_, and O_3_. Redox-active pollutants undergo chemical reactions that yield O_2_^•–^, a transient species with a short lifetime.^4,42^ Antioxidant defense molecules in the ELF, such as SOD and ascorbate, subsequently transform O_2_^•–^ into hydrogen peroxide (H_2_O_2_).^28,38^ Because H_2_O_2_ is much more stable than O_2_^•–^, it can diffuse through cell membranes and tissues.^43^ H_2_O_2_ concentrations in the ELF are tightly controlled by enzymatic processes.^4,38^ Transition metals in polluted air compete with the antioxidant defense system and facilitate the conversion of H_2_O_2_ to ^•^OH through Fenton chemistry.^4,28^ Earlier, it was shown that the ^•^OH yield through the H_2_O_2_–Fenton pathway strongly depends on the ambient PM_2.5_ concentration.^4^^•^OH is highly noxious and a main driver of oxidative stress because of its unspecific and high reactivity with all biomolecules.^5,44^

Exhaled ^•^NO mixing ratios typically range from 2 to 20 ppb in healthy and 20 to 150 ppb in diseased individuals (Table 1). The eNO concentration closely reflects the ^•^NO concentration in the ELF through a fast adsorption and desorption equilibrium governed by Henry’s law. ^•^NO (*k* ≈ 10^9^ M^–1^ s^–1^) competes with SOD (*k* ≈ 10^9^ M^–1^ s^–1^) and ascorbate (*k* ≈ 10^4^ M^–1^ s^–1^) for O_2_^•–^ consumption. ^•^NO reacts with O_2_^•–^ to form ONOO^–^, which, in turn, can react with CO_2_, ascorbate, and thiols, but also decomposes to form ^•^OH after protonation to peroxynitrous acid.^22,24,45^ Comparison of ^•^OH yields from the two competing O_2_^•–^ loss pathways, i.e., the H_2_O_2_–Fenton pathway and the ONOO^–^ pathway, is indicative of whether increased levels of ^•^NO alleviate or aggravate pollution-induced oxidative stress.

**Table 1:** Compilation of Studies Presenting Exhaled ^•^NO Concentrations (eNO), with the Corresponding Calculated Gross ^•^OH Production (*P*_OH_) Resulting from the Standard Air Pollution in Groups of Diseased Individuals and Their Respective Control Groups^a^

**eNO (ppb)**		**ΔeNO**	***P***_**OH**_**(nmol L**^–**1**^**)**				
**controls**	**patients**	**(fold change)**	**controls**	**patients**	**Δ*****P***_**OH**_**(%)**	disease	**refs**
9	26^†^	2.9	18.77	20.38	8.59	asthma	(65)
23	32^†^	1.4	20.12	20.86	3.69	asthma	(65)
4.7	13.2	2.8	18.29	19.20	4.98	asthma	(66)
7	40.5	5.8	18.55	21.48	15.80	asthma	(66)
80.2	283	3.5	23.73	28.72	21.01	asthma	(18)
9	39	4.3	18.77	21.37	13.89	asthma	(67)
18^†^	51	2.8	19.67	22.17	12.73	asthma	(68)
17	54.2	3.2	19.57	22.37	14.29	asthma	(69)
4.8	13.3	2.8	18.30	19.21	4.97	asthma	(70)
2	8.5	4.3	17.97	18.71	4.11	asthma	(13)
4.8	11.7	2.4	18.30	19.05	4.09	rhinitis	(70)
4.8	19	4.0	18.30	19.76	7.97	allergenic asthma	(70)
88	162	1.8	24.08	26.53	10.17	allergenic asthma	(71)
4.8	14.3	3.0	18.30	19.31	5.51	allergenic asthma + rhinitis	(70)
88	113	1.3	24.08	25.05	4.03	asthma	(71)
9	65	7.2	18.77	22.97	22.41	eosinophilic bronchitis	(67)
2	12	6.0	17.97	19.08	6.16	eosinophilic bronchitis	(13)
9.7	25.7	2.7	18.84	20.35	8.02	COPD	(16)
9.4	15.1	1.6	18.81	19.39	3.09	COPD	(72)
18^†^	28	1.6	19.67	20.54	4.44	COPD	(68)
23.8	51.1	2.1	20.19	22.17	9.85	acute lung graft rejection	(73)
9	28	3.1	18.77	20.54	9.43	median over all studies, neglecting cohort size	this study
20.8	51.3	2.5	19.93	22.19	11.36	mean over all studies, neglecting cohort size	this study

a Differences in ^•^NO concentrations may result from differences in sampling techniques and setups.^18,64^ In some studies, ^•^NO concentration ranges are given instead of averages. From these studies, the average value was inferred from the available data and marked with ^†^.

**Figure 2:**
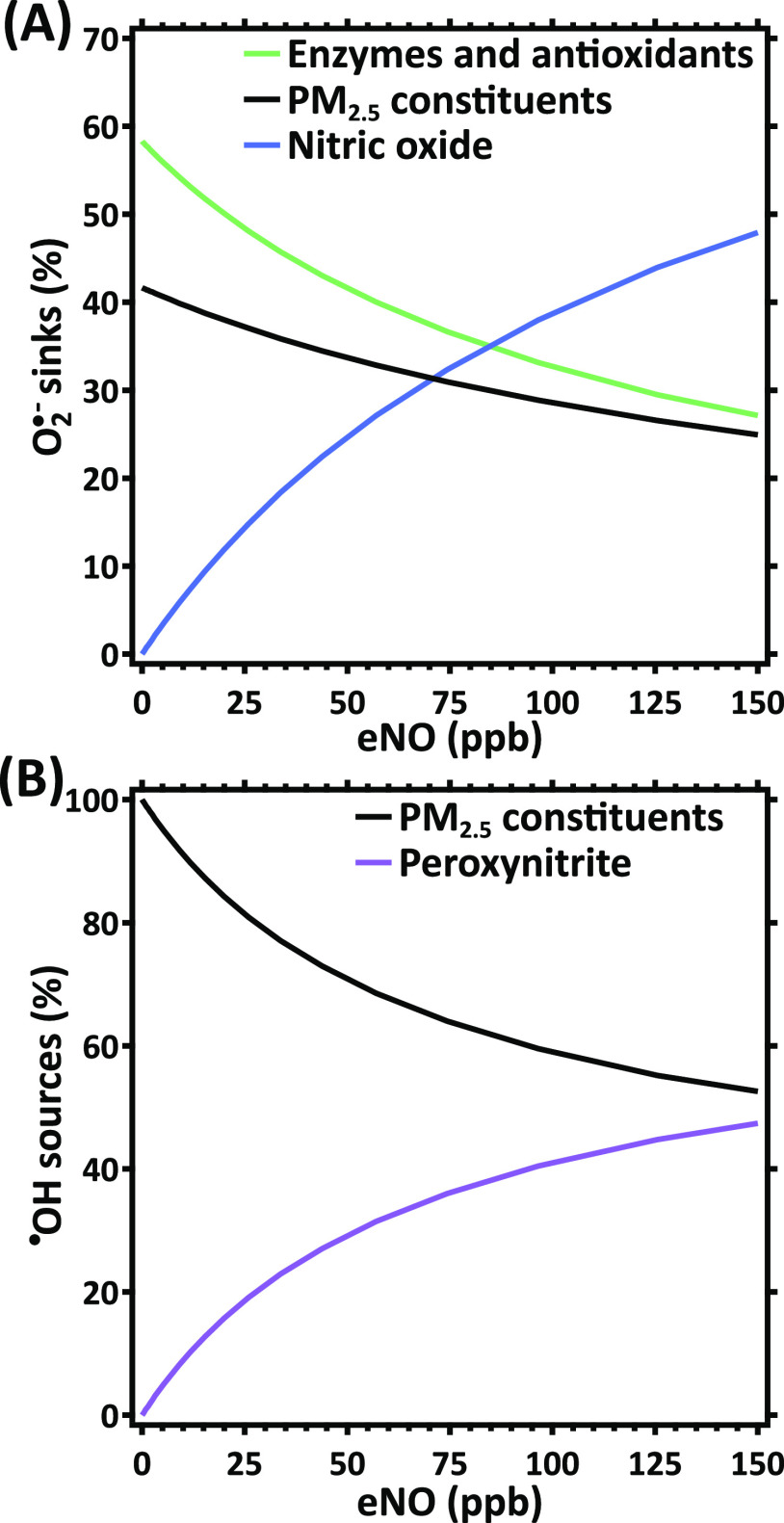
Effect of endogenous ^•^NO on the ELF redox chemistry. The main (A) reaction pathways of O_2_^•–^ and (B) sources of ^•^OH are displayed as a function of the exhaled ^•^NO mixing ratio (eNO). In (A) and (B), ‘PM_2.5_ constituents’ include copper and iron ions, SOA, and quinones, while “enzymes and antioxidants” refer to SOD and ascorbate, respectively. Calculations are performed using the standard air pollution scenario.

In Figure 2A, the competing reactions of O_2_^•–^ are presented as a function of eNO. The calculations assume an air pollution scenario of 20 μg m^–3^ PM_2.5_ and ^•^NO_2_, as well as 30 ppb O_3_, which we refer to as “standard air pollution scenario” in this study. Note that this PM_2.5_ mass concentration is four times the latest WHO guideline for the annual average concentration and double the recommended ambient air quality annual standards in the USA, Australia, and Canada. The composition of PM_2.5_ represents median mass fractions of copper and iron ions, quinones, and secondary organic aerosol from a wide range of field measurements reported in the literature (Tables S2–S4).^4^ In the absence of ^•^NO, about 60% of the O_2_^•–^ produced by air pollutants reacts with enzymes and antioxidants, while 40% reacts with PM_2.5_ constituents. With increasing eNO, O_2_^•–^ progressively reacts with ^•^NO to form ONOO^–^. This reaction accounts for about 10% of the O_2_^•–^ reactivity at an eNO of 20 ppb and for about 50% at an eNO of 150 ppb.

Figure 2B shows the main sources of ^•^OH in the ELF as a function of eNO. In the absence of ^•^NO, PM_2.5_ constituents dominate ^•^OH formation through Fenton and Fenton-like reactions, i.e., reactions of iron and copper ions with peroxides. With increasing eNO, however, decomposition of ONOO^–^ becomes a more significant source of ^•^OH and causes 15% of ^•^OH production in individuals exhaling 20 ppb ^•^NO and almost 50% in those exhaling 150 ppb.

**Figure 3:**
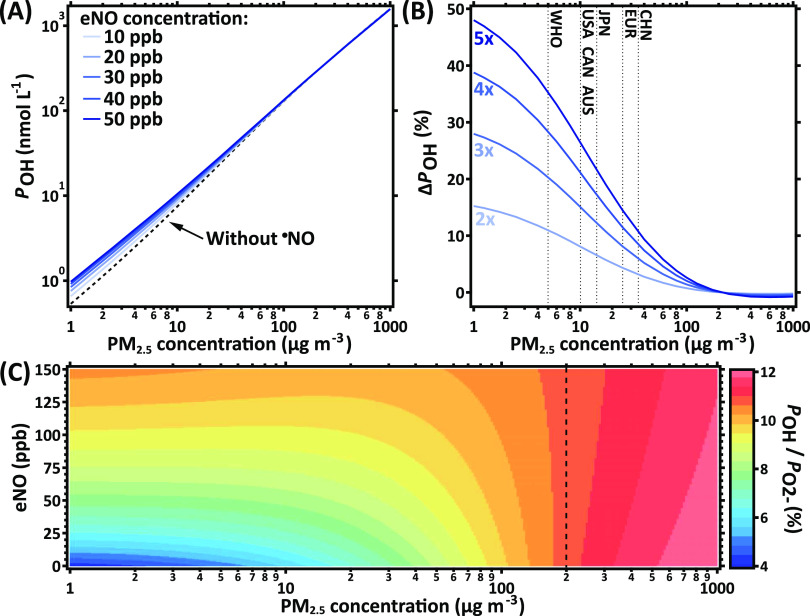
Role of endogenous ^•^NO is most significant at low PM_2.5_ concentrations. (A) Gross chemical ^•^OH production, *P*_OH_, as a function of PM_2.5_ concentration calculated for different exhaled ^•^NO concentrations. (B) The change in *P*_OH_ from an *x*-fold increase in eNO over a mixing ratio of 10 ppb is typically seen in healthy controls. (C) The ^•^OH yield as a function of PM_2.5_ and eNO concentrations. PM_2.5_ with standard composition was used in the calculations as described in the main text and presented in Tables S2–S4. The dotted lines in (B) indicate the WHO guideline and air quality standards in the United States, Canada, Australia, Japan, the European Union, and China for the annual mean PM_2.5_ concentration. The dashed vertical line in (C) indicates the PM_2.5_ concentration above which ^•^NO decreases the ^•^OH yield.

In Figure 3A, the gross chemical ^•^OH production, *P*_OH_, in nmol L^–1^ is shown for different exhaled ^•^NO concentrations and presented as a function of the ambient PM_2.5_ mass concentration. The eNO concentrations are based on observational data (Table 1) and are typical for healthy and diseased individuals. *P*_OH_ increases almost linearly with PM_2.5_ concentration independent of the eNO concentration. Higher eNO concentrations are associated with significantly higher *P*_OH_ at PM_2.5_ concentrations below 50 μg m^–3^, while the effect of ^•^NO is minor at high PM_2.5_. Hence, elevated ^•^NO concentrations can not only shift the pathways of O_2_^•–^ consumption and ^•^OH formation but also increase ^•^OH production.

Figure 3B shows the relative change in *P*_OH_, Δ*P*_OH_, between individuals exhaling 2–5 times elevated ^•^NO above a healthy eNO baseline of 10 ppb as a function of PM_2.5_ concentration. At PM_2.5_ concentrations below the WHO guideline limit of 5 μg m^–3^, individuals exhaling elevated ^•^NO experience 10–50% more ^•^OH production compared to healthy individuals. At the EU ambient air quality standard PM_2.5_ reference concentration, which is 25 μg m^–3^, the ^•^OH production in diseased individuals still increases by about 5 to 15%. Note that, at PM_2.5_ concentrations in excess of 200 μg m^–3^, higher eNO values result in lower *P*_OH_ and thus negative Δ*P*_OH_ values.

This finding can be understood by studying the eNO- and PM_2.5_-dependent yield of ^•^OH production from O_2_^•–^ in KM-SUB-ELF, which is shown in Figure 3C. At low PM_2.5_ concentrations, the ^•^OH yield is strongly dependent on eNO, as indicated by horizontal contour lines. At a PM_2.5_ concentration of ∼200 μg m^–3^ and an ^•^OH yield of around 10%, this dependence reverses as contour lines become vertical, which is highlighted with a black dashed line to guide the eye. Note that the ONOO^–^ pathway of ^•^OH production from O_2_^•–^ has a yield of 10% in the model and is independent of PM_2.5_ or eNO concentration (Figure S1). The ^•^OH yield through the H_2_O_2_–Fenton pathway, however, is strongly dependent on ambient PM_2.5_ because at increasing concentrations of transition metals in the ELF, conversion of H_2_O_2_ into ^•^OH becomes increasingly dominant over scavenging of H_2_O_2_ by catalase.^4^ The reversal of the eNO concentration dependence of ^•^OH production is thus observed when the ^•^OH yield of the H_2_O_2_–Fenton pathway surpasses the ^•^OH yield of the ONOO^–^ pathway. It is noted, however, that it occurs at an ^•^OH yield slightly higher than 10% because SOA from PM_2.5_ and O_3_ reacting with surfactants results in O_2_^•–^-independent H_2_O_2_ and ^•^OH production. Note also that during long-term exposure, eNO and PM2.5 concentrations will not necessarily remain independent variables, as discussed further below.

**Figure 4:**
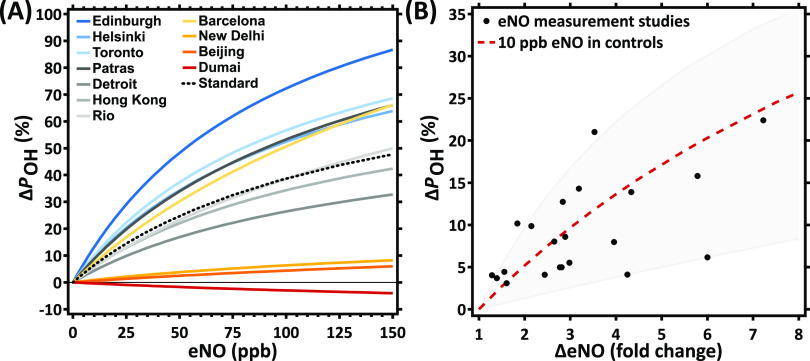
Relative change in gross chemical ^•^OH production, Δ*P*_OH_, as a function of eNO. In (A), the different air pollution scenarios are representative of a range of geographical locations. Lines are color-coded according to the PM_2.5_ mass concentration at each location (Table 2), where blue, gray, and red shades indicate low, medium, and high PM_2.5_, respectively. In (B), Δ*P*_OH_ is a function of the relative change in eNO, ΔeNO, reported in observational studies (Table 1). For each observational study, a marker represents the *P*_OH_ increase corresponding to the eNO increase between the diseased individuals and their respective control group (Table 1). All calculations for (B) are performed using the standard air pollution scenario.

Figure 4A illustrates the change in gross chemical ^•^OH production, Δ*P*_OH_, resulting from endogenous ^•^NO for a range of explicit air pollution scenarios that are representative of many geographical locations (Table 2). As mentioned above, the effect of ^•^NO on ^•^OH production is strongest at locations with a relatively low PM_2.5_ mass concentration. For example, in individuals exhaling 50 ppb ^•^NO in air with characteristics typical for Edinburgh, *P*_OH_ is increased by around 50% compared to a hypothetical case of 0 ppb eNO, whereas those exposed to air pollution typical for Beijing would only experience an increase of around 2%, as ^•^OH production is overtaken by alternate pathways. Note that the Beijing scenario has a PM_2.5_ concentration very close to but underneath the point where the model predicts a reversal of the ^•^NO effect (∼200 μg m^–3^). Conversely, a peat fire episode in Dumai, Indonesia, led to very high local PM_2.5_ concentrations, exceeding concentrations of 600 μg m^–3^ (Table 2), at which the model predicts a decrease of ^•^OH production in the ELF with increasing eNO. Furthermore, we find that Δ*P*_OH_ is influenced by the PM_2.5_ mass concentration and PM_2.5_ composition (Figure S2). For example, despite a higher PM_2.5_ mass concentration, the Barcelona scenario leads to a larger Δ*P*_OH_ than the Detroit scenario. The finding that the pollutant concentration shows a strong influence on Δ*P*_OH_ may be reinforced further by a similar effect that is attributed to ambient ^•^NO concentrations. Figure S4 shows that higher ambient ^•^NO concentrations, similar to what we find for a high PM2.5 mass concentration, dampen the effect of eNO on Δ*P*_OH_.

**Table 2:** Sampling Location, PM_2.5_ Mass Concentration, and PM_2.5_ Transition Metal Mass Fractions (MF), Including the Corresponding Calculated Gross Chemical ^•^OH Productions (*P*_OH_) without eNO and with 50 ppb eNO^a^

**sampling location**	**PM**_**2.5**_	**Fe MF**	**Cu MF**	***P***_**OH**_**(nmol L**^–**1**^**)**		Δ***P***_**OH**_
**(country)**	**(μg m**^–**3**^**)**	**(unitless)**	**(unitless)**	**at 0 ppb eNO**	**at 50 ppb eNO**	**(%)**
Edinburgh (GBR)	7.1	3.89 × 10^–3^	1.96 × 10^–4^	3.49	5.18	48.08
Helsinki (FIN)	11.8	8.14 × 10^–3^	2.63 × 10^–4^	9.00	12.07	34.16
Toronto (CAN)	12.7	4.33 × 10^–3^	1.97 × 10^–4^	6.97	9.56	37.16
Patras (GRC)	17.4	7.13 × 10^–3^	4.18 × 10^–4^	14.34	19.19	33.82
Detroit (USA)	23.0	1.02 × 10^–2^	2.61 × 10^–4^	24.13	28.18	16.82
Hong Kong (HKG)	29.0	4.83 × 10^–3^	1.97 × 10^–4^	19.31	23.55	21.96
Rio de Janeiro (BRA)	29.2	1.05 × 10^–2^	1.20 × 10^–3^	44.58	54.90	23.15
Barcelona (ESP)	35.0	7.43 × 10^–3^	1.49 × 10^–3^	45.68	59.43	30.11
New Delhi (IND)	58.2	1.22 × 10^–2^	3.44 × 10^–4^	91.61	95.10	3.81
Beijing (CHN)	182.2	6.50 × 10^–3^	3.84 × 10^–4^	223.86	229.42	2.49
Dumai (IDN)	640.0	7.52 × 10^–3^	1.56 × 10^–4^	867.78	853.15	–1.69
*standard scenario*	20.0	*8.14 × 10*^–*3*^	*3.07 × 10*^–*4*^	17.73	20.70	16.74

a The PM_2.5_ transition metal mass fractions of the standard scenario are from ref (4) and are based on the median mass fractions from a wide range of field measurements from the literature (Tables S2–S4).

The impact of elevated ^•^NO on oxidative stress in different states of health is shown in Figure 4B. Exhaled ^•^NO measurements on healthy and diseased subjects vary strongly among studies (Table 1), and especially the choice of ^•^NO sampling technique may lead to large and systematic differences.^46^ To account for such systematic differences, we use here the fold change increase in eNO from healthy to diseased states (ΔeNO) in individual studies. The increase in *P*_OH_ (Δ*P*_OH_) between healthy and diseased groups is calculated according to the standard pollution scenario and plotted against ΔeNO as black markers in Figure 4B. We find that Δ*P*_OH_ depends primarily on ΔeNO but secondarily on the baseline levels in healthy controls, with relatively high (30 ppb) and low (2 ppb) healthy-state eNO values, leading to Δ*P*_OH_ at the upper and lower ends of the gray-shaded area, respectively. The central red dashed line represents 10 ppb eNO in healthy controls. For the majority of observational studies, the model suggests that diseased subjects would experience between 5 and 15% more ^•^OH production compared to healthy subjects. Note that antioxidant concentrations in the ELF may also change with disease state,^47,48^ which is explored in Figure S5. At low antioxidant concentrations, the relative effect of eNO on Δ*P*_OH_ is further increased in the model due to less efficient transition metal recycling and a lower ^•^OH yield of the H_2_O_2_–Fenton pathway. Thus, decreased antioxidant levels may exacerbate an ^•^NO-mediated inflammation–oxidative stress feedback.

This study demonstrates a chemical mechanism through which diseased individuals may experience higher ^•^OH production and thus oxidative stress from exposure to most air pollution scenarios. Only at very high PM_2.5_ concentrations do we observe a limitation or even reversal of this effect. The point at which the effect of ^•^NO reverses depends on the yields of two major pathways from O_2_^•–^ to ^•^OH, i.e., the H_2_O_2_–Fenton and ONOO^–^ pathways of ^•^OH formation, both of which are inferred from chemical reaction rate coefficients reported in the literature and thus subject to uncertainty. The ^•^OH yield of the ONOO^–^ pathway of ^•^OH formation, for example, is sensitive to the sink reactions of ONOO^–^. The major ONOO^–^ sinks included in this study are antioxidants (i.e., glutathione, ascorbic acid, and uric acid), the enzyme glutathione peroxidase, and especially the very abundant respiratory gas CO_2_ (Table S1 and Figure S1). The reaction of ONOO^–^ with CO_2_ leads to the carboxyl radical (CO_3_^•–^) formation, which may lead to further adverse health effects, as it has been suggested to promote protein nitration reactions.^22,45^ Furthermore, the reaction between ^•^NO_2_ and ^•^NO leads to the potent nitrosylation agent N_2_O_3_. The formation of such species may add to the oxidative burden posed by elevated ^•^NO levels in diseased individuals and is supported by an earlier study that found increased ^•^NO-dependent metabolites in the ELF of asthmatic children.^49^ Hence, the downstream effects of CO_3_^•–^ and N_2_O_3_, particularly on protein oxidation and nitration,^32^ warrant further investigation.

It was shown previously that activation of inducible nitric oxide synthase (iNOS), and thus the production of ^•^NO,^50^ plays a critical role in the development of tobacco-smoke-induced pulmonary hypertension,^51,52^ a condition preceding COPD.^53^ Moreover, it has been suggested that PM_2.5_ exposure activates iNOS, which causes elevated levels of ^•^NO and is related to an increase in inflammatory and oxidative markers in the lungs of mice.^54^ A feedback between pollutant exposure, iNOS activation, and elevated ^•^NO concentrations in the airways is not considered in this study’s simulations of short-term exposure to air pollution. Nevertheless, our calculations provide a mechanistic rationale of the causal link between elevated ^•^NO, pollutant exposure, and the development of disease, as suggested by these earlier studies, and are consistent with a relatively strong relationship between PM_2.5_ and health outcomes, even at low ambient concentrations.^55^ Furthermore, a recent study showed that activated macrophages may also produce O_2_^•–^ through NADPH oxidase (NOX) enzymes in response to exposure to PM_2.5_ constituents.^31^ Higher levels of O_2_^•–^ in the ELF may further increase the importance of ONOO^–^ as a source of ^•^OH compared to other reaction pathways and may exacerbate the effect of ^•^NO. Activation of iNOS and NOX are two examples of indirect, induced effects of exposure to air pollution that might trigger positive reinforcement loops, which should be the subject of future studies.

This study shows that endogenous ^•^NO contributes to oxidative stress in the ELF, and it presents a mechanism that may explain the increased susceptibility of diseased individuals to air pollution. Chemical reactions of air pollutants result in the production and interconversion of reactive species in the ELF, including O_2_^•–^, H_2_O_2_, and ^•^OH. Their concentrations are tightly controlled by antioxidant defenses in the ELF, but transition metals from PM_2.5_ induce Fenton(-like) reactions that promote the formation of ^•^OH radicals.^4,28,56^ Endogenous ^•^NO competes with antioxidant defenses for O_2_^•–^ and causes the formation of ONOO^–^, which can decompose and form ^•^OH. Thus, the presence of ^•^NO in the ELF redirects chemical fluxes of ROS and may increase ^•^OH production from inhaled air pollutants. The noxious ^•^OH radicals readily react with biomolecules, contributing to damage-associated molecular patterns that trigger a positive feedback cycle of oxidative stress, inflammation, and disease,^6,57−59^ that further increases ^•^NO levels. While this kinetic modeling study provides a framework and first quantitative estimates of the effects of endogenous ^•^NO on the redox chemistry of air pollutants in the ELF, further experimental research will be needed to confirm or refute the exploratory results presented here. Besides fully resolving the complex interplay of the endogenous and pollution-related species, processes, and effects outlined above, future studies should aim to consider additional air pollutants such as mineral dust,^60,61^ inorganic acids and salts,^62^ polycyclic aromatic hydrocarbons (PAH),^63^ and their metabolites.

## Supporting Information Available

Overview KM-SUB-ELF (S1); exhaled ^•^NO (S2); particulate pollutant concentrations in the ELF (S3); parameterization of ROS formation from secondary organic aerosol (S4); antioxidant concentrations (S5); enzyme concentrations and kinetics (S6); chemical reactions and rate constants as used in KM-SUB-ELF (Table S1); transition metal concentrations and mass fractions in PM_2.5_ (Table S2); secondary organic aerosol (SOA) concentrations and mass fractions in PM_2.5_ (Table S3); phenanthrenequinone (PQN), 1,4-naphthoquinone (1,4-NQN), and 1,2-naphthoquinone (1,2-NQN) concentrations and mass fractions in PM_2.5_ (Table S4); products of peroxynitrite and peroxynitrous acid loss reactions (Figure S1); sensitivity of *P*_OH_ on PM_2.5_ mass and transition metal mass fraction (Figure S2); schematic overview and principal model inputs and typical model outputs of KM-SUB-ELF (Figure S3); sensitivity of *P*_OH_ to ambient ^•^NO (Figure S4); and effect of decreased and increased antioxidant levels on ^•^OH production (Figure S5) (PDF)

## References

[ref1] Burnett R.; Chen H.; Szyszkowicz M.; Fann N.; Hubbell B.; Pope C. A.; Apte J. S.; Brauer M.; Cohen A.; Weichenthal S.; Coggins J.; Di Q.; Brunekreef B.; Frostad J.; Lim S. S.; Kan H.; Walker K. D.; Thurston G. D.; Hayes R. B.; Lim C. C.; Turner M. C.; Jerrett M.; Krewski D.; Gapstur S. M.; Diver W. R.; Ostro B.; Goldberg D.; Crouse D. L.; Martin R. V.; Peters P.; Pinault L.; Tjepkema M.; van Donkelaar A.; Villeneuve P. J.; Miller A. B.; Yin P.; Zhou M.; Wang L.; Janssen N. A. H.; Marra M.; Atkinson R. W.; Tsang H.; Quoc Thach T.; Cannon J. B.; Allen R. T.; Hart J. E.; Laden F.; Cesaroni G.; Forastiere F.; Weinmayr G.; Jaensch A.; Nagel G.; Concin H.; Spadaro J. V. Global Estimates of Mortality Associated with Long-Term Exposure to Outdoor Fine Particulate Matter. Proc. Natl. Acad. Sci. U.S.A. 2018, 115 (38), 9592–9597. 10.1073/pnas.1803222115. 30181279 PMC6156628

[ref2] Landrigan P. J.; Fuller R.; Acosta N. J. R.; Adeyi O.; Arnold R.; Basu N. Nil.; Baldé A. B.; Bertollini R.; Bose-O’Reilly S.; Boufford J. I.; Breysse P. N.; Chiles T.; Mahidol C.; Coll-Seck A. M.; Cropper M. L.; Fobil J.; Fuster V.; Greenstone M.; Haines A.; Hanrahan D.; Hunter D.; Khare M.; Krupnick A.; Lanphear B.; Lohani B.; Martin K.; Mathiasen K. V.; McTeer M. A.; Murray C. J. L.; Ndahimananjara J. D.; Perera F.; Potočnik J.; Preker A. S.; Ramesh J.; Rockström J.; Salinas C.; Samson L. D.; Sandilya K.; Sly P. D.; Smith K. R.; Steiner A.; Stewart R. B.; Suk W. A.; van Schayck O. C. P.; Yadama G. N.; Yumkella K.; Zhong M. The Lancet Commission on Pollution and Health. Lancet 2018, 391 (10119), 462–512. 10.1016/S0140-6736(17)32345-0. 29056410

[ref3] Shiraiwa M.; Ueda K.; Pozzer A.; Lammel G.; Kampf C. J.; Fushimi A.; Enami S.; Arangio A. M.; Fröhlich-Nowoisky J.; Fujitani Y.; Furuyama A.; Lakey P. S. J.; Lelieveld J.; Lucas K.; Morino Y.; Pöschl U.; Takahama S.; Takami A.; Tong H.; Weber B.; Yoshino A.; Sato K. Aerosol Health Effects from Molecular to Global Scales. Environ. Sci. Technol. 2017, 51 (23), 13545–13567. 10.1021/acs.est.7b04417. 29111690

[ref4] Lelieveld S.; Wilson J.; Dovrou E.; Mishra A.; Lakey P. S. J.; Shiraiwa M.; Pöschl U.; Berkemeier T. Hydroxyl Radical Production by Air Pollutants in Epithelial Lining Fluid Governed by Interconversion and Scavenging of Reactive Oxygen Species. Environ. Sci. Technol. 2021, 55 (20), 14069–14079. 10.1021/acs.est.1c03875. 34609853 PMC8529872

[ref5] Sies H.; Berndt C.; Jones D. P. Oxidative Stress. Annu. Rev. Biochem. 2017, 86, 715–748. 10.1146/annurev-biochem-061516-045037. 28441057

[ref6] Al-Kindi S. G.; Brook R. D.; Biswal S.; Rajagopalan S. Environmental Determinants of Cardiovascular Disease: Lessons Learned from Air Pollution. Nat. Rev. Cardiol. 2020, 17 (10), 656–672. 10.1038/s41569-020-0371-2. 32382149 PMC7492399

[ref7] Guarnieri M.; Balmes J. R. Outdoor Air Pollution and Asthma. Lancet 2014, 383 (9928), 1581–1592. 10.1016/S0140-6736(14)60617-6. 24792855 PMC4465283

[ref8] Li J.; Sun S.; Tang R.; Qiu H.; Huang Q.; Mason T.; Tian L. Major Air Pollutants and Risk of COPD Exacerbations: A Systematic Review and Meta-Analysis. Int. J. Chronic Obstruct. Pulm. Dis. 2016, 11, 3079–3091. 10.2147/COPD.S122282. PMC5161337 28003742

[ref9] Goss C. H.; Newsom S. A.; Schildcrout J. S.; Sheppard L.; Kaufman J. D. Effect of Ambient Air Pollution on Pulmonary Exacerbations and Lung Function in Cystic Fibrosis. Am. J. Respir. Crit. Care Med. 2004, 169 (7), 816–821. 10.1164/rccm.200306-779OC. 14718248

[ref10] Sacks J. D.; Stanek L. W.; Luben T. J.; Johns D. O.; Buckley B. J.; Brown J. S.; Ross M. Particulate Matter–Induced Health Effects: Who Is Susceptible?. Environ. Health Perspect. 2011, 119 (4), 446–454. 10.1289/ehp.1002255. 20961824 PMC3080924

[ref11] Hooper L. G.; Kaufman J. D. Ambient Air Pollution and Clinical Implications for Susceptible Populations. Ann. Am. Thorac. Soc. 2018, 15, 5 10.1513/AnnalsATS.201707-574MG. PMC5955035 29676646

[ref12] Lundberg J. O. N.; Weitzberg E.; Lundberg J. M.; Alving K. Nitric Oxide in Exhaled Air. Eur. Respir. J. 1996, 9 (12), 2671–2680. 10.1183/09031936.96.09122671. 8980984

[ref13] Brightling C. E. Comparison of Airway Immunopathology of Eosinophilic Bronchitis and Asthma. Thorax 2003, 58 (6), 528–532. 10.1136/thorax.58.6.528. 12775868 PMC1746707

[ref14] Hamid Q.; Springall Dr.; Polak J.; Riveros-Moreno V.; Chanez P.; Bousquet J.; Godard P.; Holgate S.; Howarth P.; Redington A. Induction of Nitric Oxide Synthase in Asthma. Lancet 1993, 342 (8886–8887), 1510–1513. 10.1016/S0140-6736(05)80083-2. 7504773

[ref15] Lane C. Epithelial Inducible Nitric Oxide Synthase Activity Is the Major Determinant of Nitric Oxide Concentration in Exhaled Breath. Thorax 2004, 59 (9), 757–760. 10.1136/thx.2003.014894. 15333851 PMC1747143

[ref16] Corradi M.; Majori M.; Cacciani G. C.; Consigli G. F.; de’Munari E.; Pesci A. Increased Exhaled Nitric Oxide in Patients with Stable Chronic Obstructive Pulmonary Disease. Thorax 1999, 54 (7), 572–575. 10.1136/thx.54.7.572. 10377199 PMC1745512

[ref17] Jatakanon A.; Lim S.; Kharitonov S. A.; Chung K. F.; Barnes P. J. Correlation between Exhaled Nitric Oxide, Sputum Eosinophils, and Methacholine Responsiveness in Patients with Mild Asthma. Thorax 1998, 53 (2), 91–95. 10.1136/thx.53.2.91. 9624291 PMC1758706

[ref18] Kharitonov S. A.; Yates D.; Robbins R. A.; Barnes P. J.; Logan-Sinclair R.; Shinebourne E. A. Increased Nitric Oxide in Exhaled Air of Asthmatic Patients. Lancet 1994, 343 (8890), 133–135. 10.1016/S0140-6736(94)90931-8. 7904001

[ref19] Farah C.; Michel L. Y. M.; Balligand J.-L. Nitric Oxide Signalling in Cardiovascular Health and Disease. Nat. Rev. Cardiol. 2018, 15 (5), 292–316. 10.1038/nrcardio.2017.224. 29388567

[ref20] Taylor E. L.; Megson I. L.; Haslett C.; Rossi A. G. Nitric Oxide: A Key Regulator of Myeloid Inflammatory Cell Apoptosis. Cell Death Differ. 2003, 10 (4), 418–430. 10.1038/sj.cdd.4401152. 12719719

[ref21] Pacher P.; Beckman J. S.; Liaudet L. Nitric Oxide and Peroxynitrite in Health and Disease. Physiol. Rev. 2007, 87 (1), 315–424. 10.1152/physrev.00029.2006. 17237348 PMC2248324

[ref22] Radi R. Oxygen Radicals, Nitric Oxide, and Peroxynitrite: Redox Pathways in Molecular Medicine. Proc. Natl. Acad. Sci. U.S.A. 2018, 115 (23), 5839–5848. 10.1073/pnas.1804932115. 29802228 PMC6003358

[ref23] Mahoney L. R. Evidence for the Formation of Hydroxyl Radicals in the Isomerization of Pernitrous Acid to Nitric Acid in Aqueous Solution. J. Am. Chem. Soc. 1970, 92 (17), 5262–5263. 10.1021/ja00720a068.

[ref24] Radi R. Peroxynitrite, a Stealthy Biological Oxidant. J. Biol. Chem. 2013, 288 (37), 26464–26472. 10.1074/jbc.R113.472936. 23861390 PMC3772193

[ref25] Beckman J. S.; Beckman T. W.; Chen J.; Marshall P. A.; Freeman B. A. Apparent Hydroxyl Radical Production by Peroxynitrite: Implications for Endothelial Injury from Nitric Oxide and Superoxide. Proc. Natl. Acad. Sci. U.S.A. 1990, 87 (4), 1620–1624. 10.1073/pnas.87.4.1620. 2154753 PMC53527

[ref26] Hogg N.; Darley-Usmar V.; Wilson M.; Moncada S. Production of Hydroxyl Radicals from the Simultaneous Generation of Superoxide and Nitric Oxide. Biochem. J. 1992, 281 (2), 419–424. 10.1042/bj2810419. 1310595 PMC1130701

[ref27] Kumar M.; Liu G.-J.; Floyd R. A.; Grammas P. Anoxic Injury of Endothelial Cells Increases Production of Nitric Oxide and Hydroxyl Radicals. Biochem. Biophys. Res. Commun. 1996, 219 (2), 497–501. 10.1006/bbrc.1996.0262. 8605016

[ref28] Lakey P. S. J.; Berkemeier T.; Tong H.; Arangio A. M.; Lucas K.; Pöschl U.; Shiraiwa M. Chemical Exposure-Response Relationship between Air Pollutants and Reactive Oxygen Species in the Human Respiratory Tract. Sci. Rep. 2016, 6 (1), 32916 10.1038/srep32916. 27605301 PMC5015057

[ref29] Dovrou E.; Lelieveld S.; Mishra A.; Pöschl U.; Berkemeier T. Influence of Ambient and Endogenous H_2_O_2_ on Reactive Oxygen Species Concentrations and OH Radical Production in the Respiratory Tract. Environ. Sci.: Atmos 2023, 3, 1066–1074. 10.1039/D2EA00179A.

[ref30] Weichenthal S.; Shekarrizfard M.; Kulka R.; Lakey P. S. J.; Al-Rijleh K.; Anowar S.; Shiraiwa M.; Hatzopoulou M. Spatial Variations in the Estimated Production of Reactive Oxygen Species in the Epithelial Lung Lining Fluid by Iron and Copper in Fine Particulate Air Pollution. Environ. Epidemiol. 2018, 2 (3), e020 10.1097/EE9.0000000000000020. 33210071 PMC7662795

[ref31] Fang T.; Huang Y.-K.; Wei J.; Monterrosa Mena J. E.; Lakey P. S. J.; Kleinman M. T.; Digman M. A.; Shiraiwa M. Superoxide Release by Macrophages through NADPH Oxidase Activation Dominating Chemistry by Isoprene Secondary Organic Aerosols and Quinones to Cause Oxidative Damage on Membranes. Environ. Sci. Technol. 2022, 56, 17029–17038. 10.1021/acs.est.2c03987. 36394988 PMC9730850

[ref32] Mishra A.; Lelieveld S.; Pöschl U.; Berkemeier T. Multiphase Kinetic Modeling of Air Pollutant Effects on Protein Modification and Nitrotyrosine Formation in Epithelial Lining Fluid. Environ. Sci. Technol. 2023, 57, 12642–12653. 10.1021/acs.est.3c03556. 37587684 PMC10469477

[ref33] Shiraiwa M.; Pfrang C.; Poschl U. Kinetic Multi-Layer Model of Aerosol Surface and Bulk Chemistry (KM-SUB): The Influence of Interfacial Transport and Bulk Diffusion on the Oxidation of Oleic Acid by Ozone. Atmos. Chem. Phys. 2010, 10 (8), 3673–3691. 10.5194/acp-10-3673-2010.

[ref34] Charrier J. G.; Anastasio C. Impacts of Antioxidants on Hydroxyl Radical Production from Individual and Mixed Transition Metals in a Surrogate Lung Fluid. Atmos. Environ. 2011, 45 (40), 7555–7562. 10.1016/j.atmosenv.2010.12.021. PMC3223868 22125412

[ref35] Charrier J. G.; McFall A. S.; Richards-Henderson N. K.; Anastasio C. Hydrogen Peroxide Formation in a Surrogate Lung Fluid by Transition Metals and Quinones Present in Particulate Matter. Environ. Sci. Technol. 2014, 48 (12), 7010–7017. 10.1021/es501011w. 24857372 PMC4063450

[ref36] Tong H.; Arangio A. M.; Lakey P. S. J.; Berkemeier T.; Liu F.; Kampf C. J.; Brune W. H.; Pöschl U.; Shiraiwa M. Hydroxyl Radicals from Secondary Organic Aerosol Decomposition in Water. Atmos. Chem. Phys. 2016, 16 (3), 1761–1771. 10.5194/acp-16-1761-2016.

[ref37] Wang Y.; Kim H.; Paulson S. E. Hydrogen Peroxide Generation from α- and β-Pinene and Toluene Secondary Organic Aerosols. Atmos. Environ. 2011, 45 (18), 3149–3156. 10.1016/j.atmosenv.2011.02.060.

[ref38] Cantin A. M.; Fells G. A.; Hubbard R. C.; Crystal R. G. Antioxidant Macromolecules in the Epithelial Lining Fluid of the Normal Human Lower Respiratory Tract. J. Clin. Invest. 1990, 86 (3), 962–971. 10.1172/JCI114798. 2394842 PMC296816

[ref39] Gonzalez D. H.; Diaz D. A.; Baumann J. P.; Ghio A. J.; Paulson S. E. Effects of Albumin, Transferrin and Humic-like Substances on Iron-Mediated OH Radical Formation in Human Lung Fluids. Free Radical Biol. Med. 2021, 165, 79–87. 10.1016/j.freeradbiomed.2021.01.021. 33486087

[ref40] Tong H.; Lakey P. S. J.; Arangio A. M.; Socorro J.; Shen F.; Lucas K.; Brune W. H.; Pöschl U.; Shiraiwa M. Reactive Oxygen Species Formed by Secondary Organic Aerosols in Water and Surrogate Lung Fluid. Environ. Sci. Technol. 2018, 52 (20), 11642–11651. 10.1021/acs.est.8b03695. 30234977

[ref41] Cross C. E.; Louie S.; Halliwell B.; et al. Oxidants, Antioxidants, and Respiratory Tract Lining Fluids. Environ. Health. Perspect. 1994, 102 Suppl 10, 185–191. 10.1289/ehp.94102s10185. PMC1566988 7705296

[ref42] Pryor W. A. Oxy-Radicals and Related Species: Their Formation, Lifetimes, and Reactions. Annu. Rev. Physiol. 1986, 48 (1), 657–667. 10.1146/annurev.ph.48.030186.003301. 3010829

[ref43] Winterbourn C. C. Reconciling the Chemistry and Biology of Reactive Oxygen Species. Nat. Chem. Biol. 2008, 4 (5), 278–286. 10.1038/nchembio.85. 18421291

[ref44] Forman H. J.; Davies K. J. A.; Ursini F. How Do Nutritional Antioxidants Really Work: Nucleophilic Tone and Para-Hormesis versus Free Radical Scavenging in Vivo. Free Radical Biol. Med. 2014, 66, 24–35. 10.1016/j.freeradbiomed.2013.05.045. 23747930 PMC3852196

[ref45] Denicola A.; Freeman B. A.; Trujillo M.; Radi R. Peroxynitrite Reaction with Carbon Dioxide/Bicarbonate: Kinetics and Influence on Peroxynitrite-Mediated Oxidations. Arch. Biochem. Biophys. 1996, 333 (1), 49–58. 10.1006/abbi.1996.0363. 8806753

[ref46] Kharitonov S.; Alving K.; Barnes P. J. Exhaled and Nasal Nitric Oxide Measurements: Recommendations. Eur. Respir. J. 1997, 10 (7), 1683–1693. 10.1183/09031936.97.10071683. 9230267

[ref47] Dove R. E.; Leong-Smith P.; Roos-Engstrand E.; Pourazar J.; Shah M.; Behndig A. F.; Mudway I. S.; Blomberg A. Cigarette Smoke–Induced Induction of Antioxidant Enzyme Activities in Airway Leukocytes Is Absent in Active Smokers with COPD. Eur. Clin. Respir. J. 2015, 2 (1), 27837 10.3402/ecrj.v2.27837. PMC4629722 26557249

[ref48] Tunnicliffe W. S.; Harrison R.; Kelly F.; Dunster C.; Ayres J. The Effect of Sulphurous Air Pollutant Exposures on Symptoms, Lung Function, Exhaled Nitric Oxide, and Nasal Epithelial Lining Fluid Antioxidant Concentrations in Normal and Asthmatic Adults. Occup. Environ. Med. 2003, 60 (11), e15 10.1136/oem.60.11.e15. 14573726 PMC1740413

[ref49] Fitzpatrick A. M.; Brown L. A. S.; Holguin F.; Teague W. G. Levels of Nitric Oxide Oxidation Products Are Increased in the Epithelial Lining Fluid of Children with Persistent Asthma. J. Allergy Clin. Immunol. 2009, 124 (5), 990–996.e9. 10.1016/j.jaci.2009.08.039. 19895987 PMC2776730

[ref50] Wang C.; Liu C.; Lin H.; Yu C.; Chung K.; Kuo H. Increased Exhaled Nitric Oxide in Active Pulmonary Tuberculosis Due to Inducible NO Synthase Upregulation in Alveolar Macrophages. Eur. Respir. J. 1998, 11 (4), 809 10.1183/09031936.98.11040809. 9623681

[ref51] Seimetz M.; Parajuli N.; Pichl A.; Veit F.; Kwapiszewska G.; Weisel F. C.; Milger K.; Egemnazarov B.; Turowska A.; Fuchs B.; Nikam S.; Roth M.; Sydykov A.; Medebach T.; Klepetko W.; Jaksch P.; Dumitrascu R.; Garn H.; Voswinckel R.; Kostin S.; Seeger W.; Schermuly R. T.; Grimminger F.; Ghofrani H. A.; Weissmann N. Inducible NOS Inhibition Reverses Tobacco-Smoke-Induced Emphysema and Pulmonary Hypertension in Mice. Cell 2011, 147 (2), 293–305. 10.1016/j.cell.2011.08.035. 22000010

[ref52] Nathan C. Is INOS Beginning to Smoke?. Cell 2011, 147 (2), 257–258. 10.1016/j.cell.2011.09.031. 22000003

[ref53] Adler E. M. Two Ways to Breathe Easier Without INOS. Sci. Signaling 2011, 4 (195), ec288 10.1126/scisignal.4195ec288.

[ref54] Long M.-H.; Zhu X.-M.; Wang Q.; Chen Y.; Gan X.-D.; Li F.; Fu W.-L.; Xing W.-W.; Xu D.-Q.; Xu D.-G. PM2.5 Exposure Induces Vascular Dysfunction via NO Generated by INOS in Lung of ApoE–/– Mouse. Int. J. Biol. Sci. 2020, 16 (1), 49–60. 10.7150/ijbs.36073. 31892845 PMC6930374

[ref55] Weichenthal S.; Pinault L.; Christidis T.; Burnett R. T.; Brook J. R.; Chu Y.; Crouse D. L.; Erickson A. C.; Hystad P.; Li C.; Martin R. V.; Meng J.; Pappin A. J.; Tjepkema M.; van Donkelaar A.; Weagle C. L.; Brauer M. How Low Can You Go? Air Pollution Affects Mortality at Very Low Levels. Sci. Adv. 2022, 8 (39), eabo3381 10.1126/sciadv.abo3381. 36170354 PMC9519036

[ref56] Shen J.; Taghvaee S.; La C.; Oroumiyeh F.; Liu J.; Jerrett M.; Weichenthal S.; Del Rosario I.; Shafer M. M.; Ritz B.; Zhu Y.; Paulson S. E. Aerosol Oxidative Potential in the Greater Los Angeles Area: Source Apportionment and Associations with Socioeconomic Position. Environ. Sci. Technol. 2022, 56 (24), 17795–17804. 10.1021/acs.est.2c02788. 36472388 PMC9775201

[ref57] Lucas K.; Maes M. Role of the Toll Like Receptor (TLR) Radical Cycle in Chronic Inflammation: Possible Treatments Targeting the TLR4 Pathway. Mol. Neurobiol. 2013, 48 (1), 190–204. 10.1007/s12035-013-8425-7. 23436141 PMC7091222

[ref58] Ziegler K.; Kunert A. T.; Reinmuth-Selzle K.; Leifke A. L.; Widera D.; Weller M. G.; Schuppan D.; Fröhlich-Nowoisky J.; Lucas K.; Pöschl U. Chemical Modification of Pro-Inflammatory Proteins by Peroxynitrite Increases Activation of TLR4 and NF-KB: Implications for the Health Effects of Air Pollution and Oxidative Stress. Redox Biol. 2020, 37, 101581 10.1016/j.redox.2020.101581. 32739154 PMC7767743

[ref59] Daiber A.; Di Lisa F.; Oelze M.; Kröller-Schön S.; Steven S.; Schulz E.; Münzel T. Crosstalk of Mitochondria with NADPH Oxidase via Reactive Oxygen and Nitrogen Species Signalling and Its Role for Vascular Function. Br. J. Pharmacol. 2017, 174 (12), 1670–1689. 10.1111/bph.13403. 26660451 PMC5446573

[ref60] Nishita-Hara C.; Kobayashi H.; Hara K.; Hayashi M. Dithiothreitol-Measured Oxidative Potential of Reference Materials of Mineral Dust: Implications for the Toxicity of Mineral Dust Aerosols in the Atmosphere. Geohealth 2023, 7 (7), e2022GH000736 10.1029/2022GH000736. PMC10326488 37426691

[ref61] Tong H.; Lakey P. S. J.; Arangio A. M.; Socorro J.; Kampf C. J.; Berkemeier T.; Brune W. H.; Pöschl U.; Shiraiwa M. Reactive Oxygen Species Formed in Aqueous Mixtures of Secondary Organic Aerosols and Mineral Dust Influencing Cloud Chemistry and Public Health in the Anthropocene. Faraday Discuss. 2017, 200, 251–270. 10.1039/C7FD00023E. 28574563

[ref62] Fang T.; Guo H.; Zeng L.; Verma V.; Nenes A.; Weber R. J. Highly Acidic Ambient Particles, Soluble Metals, and Oxidative Potential: A Link between Sulfate and Aerosol Toxicity. Environ. Sci. Technol. 2017, 51 (5), 2611–2620. 10.1021/acs.est.6b06151. 28141928

[ref63] Jin L.; Xie J.; Wong C. K.; Chan S. K.; Abbaszade G.; Schnelle-Kreis J.; Zimmermann R.; Li J.; Zhang G.; Fu P.; Li X. Contributions of City-Specific Fine Particulate Matter (PM2. 5) to Differential in Vitro Oxidative Stress and Toxicity Implications between Beijing and Guangzhou of China. Environ. Sci. Technol. 2019, 53 (5), 2881–2891. 10.1021/acs.est.9b00449. 30730710

[ref64] Slutsky A.; Drazen J.; Silkoff P.; Gaston B.; Holden W.; Romero F. Recommendations for Standardized Procedures for the Online and Offline Measurement of Exhaled Lower Respiratory Nitric Oxide and Nasal Nitric Oxide in Adults and Children 1999. Am. J. Respir. Crit. Care Med. 1999, 160, 2104–2117. 10.1164/ajrccm.160.6.ats8-99. 10588636

[ref65] Alving K.; Weitzberg E.; Lundberg J. M. Increased Amount of Nitric Oxide in Exhaled Air of Asthmatics. Eur. Clin. Respir. J. 1993, 6 (9), 1386–137. 10.1183/09031936.93.06091368. 7507065

[ref66] Massaro A. F.; Mehta S.; Lilly C. M.; Kobzik L.; Reilly J. J.; Drazen J. M. Elevated Nitric Oxide Concentrations in Isolated Lower Airway Gas of Asthmatic Subjects. Am. J. Respir. Crit. Care Med. 1996, 153 (5), 1510–1514. 10.1164/ajrccm.153.5.8630594. 8630594

[ref67] Berlyne G. S.; Parameswaran K.; Kamada D.; Efthimiadis A.; Hargreave F. E. A Comparison of Exhaled Nitric Oxide and Induced Sputum as Markers of Airway Inflammation. J. Allergy Clin. Immunol. 2000, 106 (4), 638–644. 10.1067/mai.2000.109622. 11031333

[ref68] Ansarin K.; Chatkin J. M.; Ferreira I. M.; Gutierrez C. A.; Zamel N.; Chapman K. R. Exhaled Nitric Oxide in Chronic Obstructive Pulmonary Disease: Relationship to Pulmonary Function. Eur. Respir. J. 2001, 17 (5), 934–938. 10.1183/09031936.01.17509340. 11488329

[ref69] Kharitonov S. A.; Gonio F.; Kelly C.; Meah S.; Barnes P. J. Reproducibility of Exhaled Nitric Oxide Measurements in Healthy and Asthmatic Adults and Children. Eur. Respir. J. 2003, 21 (3), 433–438. 10.1183/09031936.03.00066903a. 12661997

[ref70] Gratziou Ch.; Lignos M.; Dassiou M.; Roussos C. Influence of Atopy on Exhaled Nitric Oxide in Patients with Stable Asthma and Rhinitis. Eur. Respir. J. 1999, 14 (4), 897 10.1034/j.1399-3003.1999.14d28.x. 10573239

[ref71] Lúdvíksdóttir D.; Janson C.; Högman M.; Hedenström H.; Björnsson E.; Boman G. Exhaled Nitric Oxide and Its Relationship to Airway Responsiveness and Atopy in Asthma. Respir. Med. 1999, 93 (8), 552–556. 10.1016/S0954-6111(99)90154-3. 10542988

[ref72] Yao Y.; Chen X.; Chen W.; Wang Q.; Fan Y.; Han Y.; Wang T.; Wang J.; Qiu X.; Zheng M.; Que C.; Zhu T. Susceptibility of Individuals with Chronic Obstructive Pulmonary Disease to Respiratory Inflammation Associated with Short-Term Exposure to Ambient Air Pollution: A Panel Study in Beijing. Sci. Total Environ. 2021, 766, 142639 10.1016/j.scitotenv.2020.142639. 33069482

[ref73] Silkoff P. E.; Caramori M.; Tremblay L.; McClean P.; Chaparro C.; Kesten S.; Hutcheon M.; Slutsky A. S.; Zamel N.; Keshavjee S. Exhaled Nitric Oxide in Human Lung Transplantation: A Noninvasive Marker of Acute Rejection. Am. J. Respir. Crit. Care Med. 1998, 157 (6), 1822–1828. 10.1164/ajrccm.157.6.9707159. 9620912

