# Supplementary material for: Endogenous Nitric Oxide Can Enhance Oxidative Stress Caused by Air Pollutants and Explain Higher Susceptibility of Individuals with Inflammatory Disorders
Source: Environ Sci Technol. 2024 Jan 18;58(4):1823–31. doi: 10.1021/acs.est.3c07010 (PMC10832043; doi:10.1021/acs.est.3c07010)
Supplement: Supplementary file 1 — es3c07010_si_001.pdf [file es3c07010_si_001.pdf]

Supporting Information for

Endogenous nitric oxide can enhance oxidative stress caused by air pollutants and explain higher susceptibility of individuals with inflammatory disorders

Steven Lelieveld,[1]* Jos Lelieveld,[2,3] Ashmi Mishra,[1] Andreas Daiber,[4,5] Andrea Pozzer,[2,3] Ulrich Pöschl,[1] Thomas Berkemeier[1]*

Affiliations:

[1]Multiphase Chemistry Department, Max Planck Institute for Chemistry; Mainz, Germany.

[2]Atmospheric Chemistry Department, Max Planck Institute for Chemistry; Mainz, Germany.

[3]The Energy, Environment and Water Research Center, Cyprus Institute; Nicosia, Cyprus.

[4]Department of Cardiology, University Medical Center of the Johannes Gutenberg University; Mainz, Germany

[5]German Center for Cardiovascular Research (DZHK), Partner Site Rhine-Main, Mainz, Germany

*Corresponding authors. Email: s.lelieveld@mpic.de, t.berkemeier@mpic.de.

<u>**Contents of Supporting Information**</u>

Additional information

S1: Overview KM-SUB-ELF

S2: Exhaled $^{\bullet}$NO

S3: Particulate pollutant concentrations in the ELF

S4: Parameterization of ROS formation from secondary organic aerosol

S5: Antioxidant concentrations

S6: Enzyme concentrations and kinetics

Tables

Table S1: Chemical reactions

Table S2: $PM_{2.5}$ and transition metal mass fractions

Table S3: $PM_{2.5}$ and Secondary Organic Aerosol (SOA) mass fractions

Table S4: $PM_{2.5}$ and quinone mass fractions

Figures

Figure S1: Products of peroxynitrite and peroxynitrous acid decomposition and sink reactions

Figure S2: Sensitivity of P_{OH} on $PM_{2.5}$ mass and transition metal mass fraction

Figure S3: Schematic overview of KM-SUB-ELF and principal model inputs and typical model outputs

Figure S4: Sensitivity of P_{OH} on ambient $^{\bullet}$NO

Figure S5: Effect of decreased and increased antioxidant levels on $^{\bullet}$OH production

S1. Overview KM-SUB-ELF

The kinetic multi-layer model of surface and bulk chemistry in the epithelial lining fluid (KM-SUB-ELF)[1] describes chemical reactions and mass transport in the lungs, at the intersection of atmospheric and physiological chemistry, with the goal of providing a chemical rationale for the adverse health effects or air pollution. A schematic overview of the model is shown in Figure S3. The temporal evolution of reactants and reaction products is calculated by solving a set of differential equations at high temporal resolution with MATLAB software using the ode23tb solver that employs TR-BDF2 to consider the stiffness of chemical mechanisms. Compared to previous publications, KM-SUB-ELF was extended by the molecules $^{\bullet}NO$ and CO_2. The gas-phase respiratory tract CO_2 concentration was assumed to be 3.8%. Table S1 shows the chemical mechanism used in this study, which includes redox chemistry of transition metals, HO_x and NO_x radical chemistry, antioxidant redox couples and redox reactions in a total of 139 reactions. Of these, 23 are gas-phase chemical reactions adopted from the Master Chemical Mechanism (MCM),[2,3] and 116 are aqueous-phase reactions within the ELF, of which six occur in the surfactant layer.

S2. Exhaled $^{\bullet}NO$

Exhaled $^{\bullet}NO$ (eNO) originates from inducible nitric oxide synthase (iNOS),[4] an enzyme that is present on macrophages, and produces $^{\bullet}NO$ from L-arginine and oxygen.[5] Humans typically exhale $^{\bullet}NO$ in concentrations that exceed those in the atmosphere. Thus, the ELF is a source of $^{\bullet}NO$ to the gas phase of the respiratory tract. In the model, ambient $^{\bullet}NO$ levels have a similar effect on P_{OH} as endogenously produced $^{\bullet}NO$, where a 10 ppb increase leads to an increase P_{OH} of about 1 nM in a standard pollution and exposure scenario in both healthy and diseased individuals (Figure S4a). Accordingly, the relative increase of P_{OH} due to eNO in diseased individuals (ΔP_{OH}) is slightly reduced at higher levels of ambient $^{\bullet}NO$ (Figure S4b). However, ambient $^{\bullet}NO$ levels are typically in the single-digit ppb range, much lower than the eNO levels found in diseased individuals. At such low levels, the effect of ambient $^{\bullet}NO$ is close to negligible. As for all volatile species, mass transfer of $^{\bullet}NO$ between the ELF and the gas phase of the respiratory tract is determined through explicit adsorption and desorption fluxes, which depend on molecular collision rates and Henry's law coefficient.[6] In our model, a constant flux of $^{\bullet}NO$ from the underlying epithelium to the ELF is fitted to achieve $^{\bullet}NO$ concentrations reported in the exhaled

breath of healthy volunteers, as well as rhinitis, COPD, bronchitis and asthma patients, as listed in Tab. 1 (‘Controls’ and ‘Patients’). It is noted that differences in measured $^{\bullet}NO$ may arise from differences in sampling technique, as detailed in Kharitomov et al. (1997). For instance, nasal breathing (NB) results in readings that may be one order of magnitude higher compared to oral breathing because of $^{\bullet}NO$ production in the sinuses.

S3. Particulate pollutant concentrations in the ELF

The ELF concentrations of redox-active $PM_{2.5}$ constituents ($C_{ELF,Y}$) are calculated as described previously,[8] using Eq. S1.

$$C_{ELF,Y} = \frac{C_{gas,PM2.5} \times Q \times t_{acc} \times f_{dep,PM2.5} \times w_Y \times S_Y}{M_Y \times V_{ELF}} \quad \text{(Eq. S1)}$$

Mass fractions (w) of redox-active $PM_{2.5}$ constituents Y are derived using field observations of approximately 70 sampling sites which have previously been reported and which are tabulated in Tabs. S2-S4.[8] The sampling sites represent a large range from pristine Amazonian rainforest air to the heavily polluted, hazy conditions in Beijing, China. For 'standard' $PM_{2.5}$ composition, we derive the median mass fractions of $PM_{2.5}$ constituents from all sampling sites. However, to show model sensitivity to $PM_{2.5}$ composition, and transition metals in particular, the reported $PM_{2.5}$ compositions are used explicitly in the calculations for Figure 4A. The data from sampling sites we used for Figure 4A are marked with an asterisk in Tab. S2. It was assumed that 45% of all the respired $PM_{2.5}$ deposits in the respiratory tract during a 2-hour exposure episode ($f_{dep,PM2.5}$). Solubilities (S) of the $PM_{2.5}$ constituents copper and iron were assumed to be 40% and 10%, respectively. The ventilation rate (Q) of the respiratory tract is calculated using a breath volume of 1.5 L and a breathing rate of 16 breaths per minute.

S4. Parameterization of ROS formation from secondary organic aerosol

Secondary organic aerosol (SOA) has been shown to form reactive oxygen species (ROS) upon dissolution in aqueous solutions, which is included in the model.[9–11] Because the exact reaction mechanism has not been fully elucidated, ROS formation by SOA in the ELF is parameterized using formation rates of H_2O_2 and $^{\bullet}OH$ based on experimental observations.[1,9,10] In this study, a first-order rate coefficient of H_2O_2 production is inferred from experimental observations using α- and β-pinene that found a 0.6% H_2O_2 mass yield from SOA,[9] following Lakey et al.[1] Tong et al. (2016) quantified $^{\bullet}OH$ production of SOA, from which we infer first- and second-order rate coefficients that reproduce a molar yield of 0.1% in the absence of iron (R137; Tab. S1), and a 1% yield of $^{\bullet}OH$ in the presence of iron (R139; Tab. S1), respectively, in the experimental data.

S5. Antioxidant concentrations

In this study, four antioxidants in the ELF are included. Ascobate, glutathione, uric acid have concentrations of 40, 108 and 200 µM in the aqueous ELF, respectively, whereas α-tocopherol has a concentration of 200 µM in the surfactant layer of the ELF.[1,8,12,13] Previous studies suggest that the antioxidant concentrations in the ELF may vary within a factor of two between healthy and diseased individuals.[14,15] For instance, the antioxidant levels in patients suffering from COPD have been found to be slightly increased compared to healthy individuals,[14] whereas asthmatics have slightly decreased antioxidant levels in their ELF.[15] Here. we perform a model sensitivity study and show the effect of antioxidant concentrations on P_{OH} at diseased and healthy eNO levels (Figure S5). We find that in diseases in which the antioxidant levels decrease (FC below 1), the relative effect of eNO becomes stronger (Figure S5). In diseases in which the antioxidant levels increase (FC above 1), the effect of eNO compounds with the transition-metal recycling effect of antioxidants thus increases the susceptibility of individuals to air pollution.

Studies using healthy volunteers suggest that even in extreme pollution scenarios, i.e. 1 ppm NO_2 for several hours, the antioxidants in the ELF do not fully deplete,[16] likely due to fast replenishment. All concentrations of antioxidants were thus kept constant in the model simulations in this study.

S6. Enzyme reactions and concentrations

The catalytic activity of enzymes in the ELF is implemented as previously described.[8] In brief, the molar concentrations of enzymes are calculated from enzyme activity in enzyme units (U), the catalytic constant, k_{cat}, and Eq. S2.

$$[\text{Enzyme}] = \frac{v_{max}}{k_{cat}} \qquad \text{(Eq. S2)}$$

One U is defined as the quantity of enzyme needed to catalyze 1 micromole of substrate per minute. In experiments quantifying U in biological samples (e.g. in the ELF), the substrate of the enzyme is kept in vast excess, thus $U = v_{max}$. k_{cat} describes the maximum number of chemical conversions a single active site (in our case a single enzyme) can carry out in a second.

In the ELF, two enzymes are mainly responsible for the interconversion and scavenging of ROS.[8,17] In a dismutation reaction, two superoxide ($^{\bullet}O_2^-$) molecules are converted into H_2O_2 and O_2 by superoxide dismutase (SOD; R127; Tab. S1). $U_{SOD,ELF}$ is 36.8 ± 2.0 U mL^{-1},[17] and $k_{cat,SOD}$ is reported to range between $10^5 – 10^6$ s^{-1}.[18,19] These values translate to a concentration range of SOD between 0.58 – 6.5 nM (~1 nM) in the ELF. Previous experiments have shown that catalase (CAT) is the most important endogenous H_2O_2 scavenger in the ELF.[17] $U_{cat,ELF}$ is 3.7 ± 0.6 U mL^{-1},[17] and $k_{cat,CAT}$ is reported to range between $10^5 – 10^6$ s^{-1}. These values translate into a molar concentration ranging from 1.3 – 24 pM (~5 pM) in the ELF. The glutathione peroxidase (GPx) concentration in the model ELF is 50 nM based on a molar mass of 2.19×10^4 g mol^{-1} and a mass concentration of 1 µg mL^{-1} in ELF.[20] We note that for the ELF, U, k_{cat}, and protein masses are difficult to determine because the ELF is very difficult to sample. We thus acknowledge that the enzyme concentrations used in this study are subject to uncertainty.

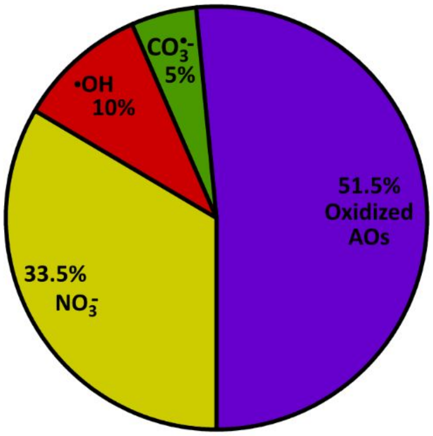

Figure S1. Relative contribution of various chemical reactions that contribute to the loss of peroxynitrite ($ONOO^-$) and peroxynitrous acid (ONOOH). "Oxidized AOs" stands for reaction partners of $ONOO^-$ that are grouped as antioxidants. Those antioxidants include glutathione, uric acid and ascorbate as well as the antioxidant enzyme glutathione peroxidase. Nitrate (NO_3^-) and $^•OH$ result from first-order decomposition of ONOOH, and the former also results from CO_2 reacting with $ONOO^-$. The carboxyl radical anion ($CO_3^{•-}$) is also a result of CO_2 reacting with $ONOO^-$.

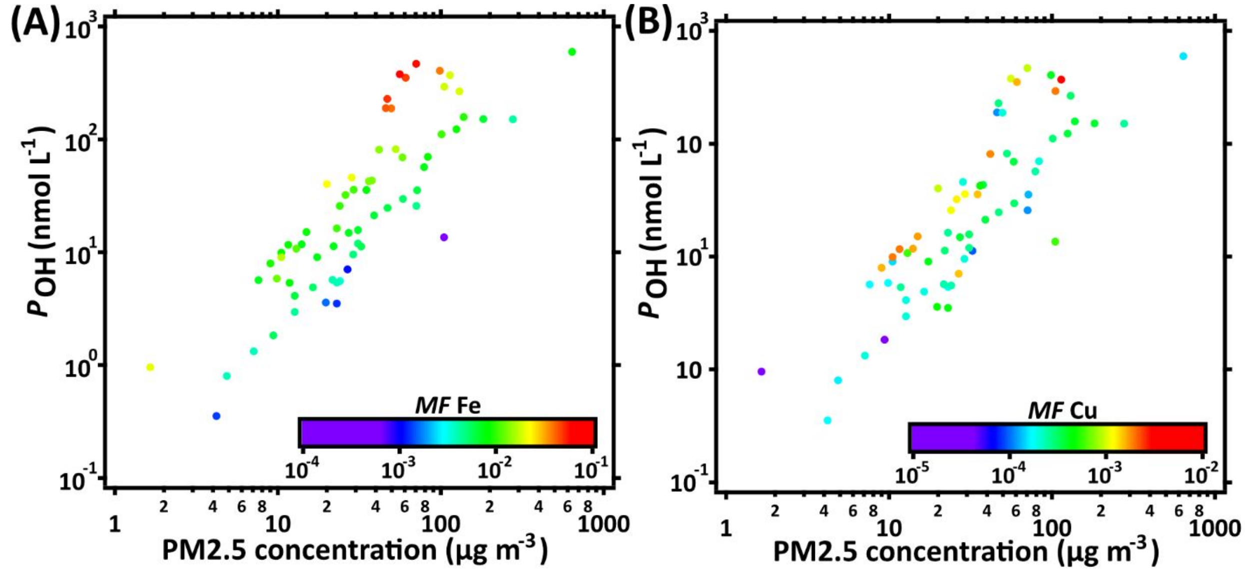

Figure S2. Gross chemical $^{\bullet}$OH production, P_{OH}, using the $PM_{2.5}$ mass and composition measured in many locations worldwide, as presented in Tab. S2. The color-coding of data points indicates the mass fractions of (**A**) iron and (**B**) copper in $PM_{2.5}$.

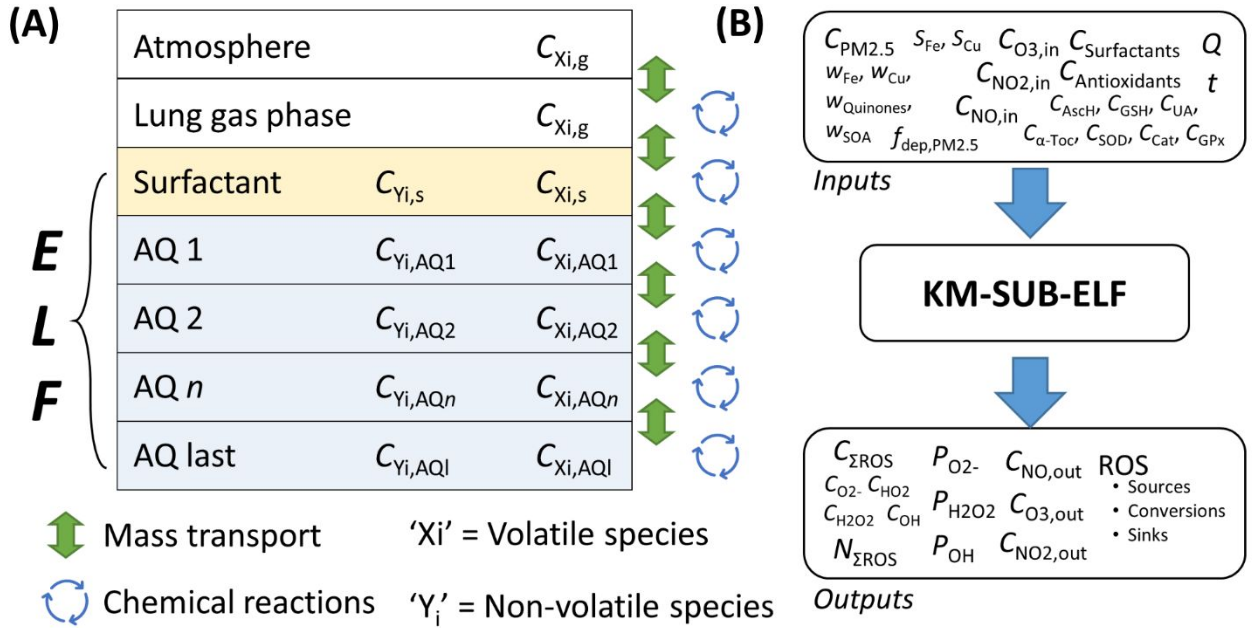

Figure S3. Schematic overview of (A) the kinetic model KM-SUB-ELF and (B) principal model inputs and typical model outputs of KM-SUB-ELF. Concentrations are indicated using *C*. $PM_{2.5}$ mass fractions of iron, copper, secondary organic aerosol and quinones are indicated using w_{Fe}, w_{Cu}, w_{SOA}, and $w_{Quinones}$, respectively. $f_{dep,PM2.5}$ represents the fraction of PM2.5 deposited in the ELF. *S* indicates the PM2.5 soluble fractions in the ELF. *Q* is the ventilation rate of the lung, and *t* the exposure time to pollutants. $N_{\Sigma ROS}$ indicates the cumulative ROS produced subtracted by ROS interconversion reactions. *P* stands for gross chemically produced. Units and additional information are supplied in the main and SI text, and in Lelieveld et al. 2021.[8]

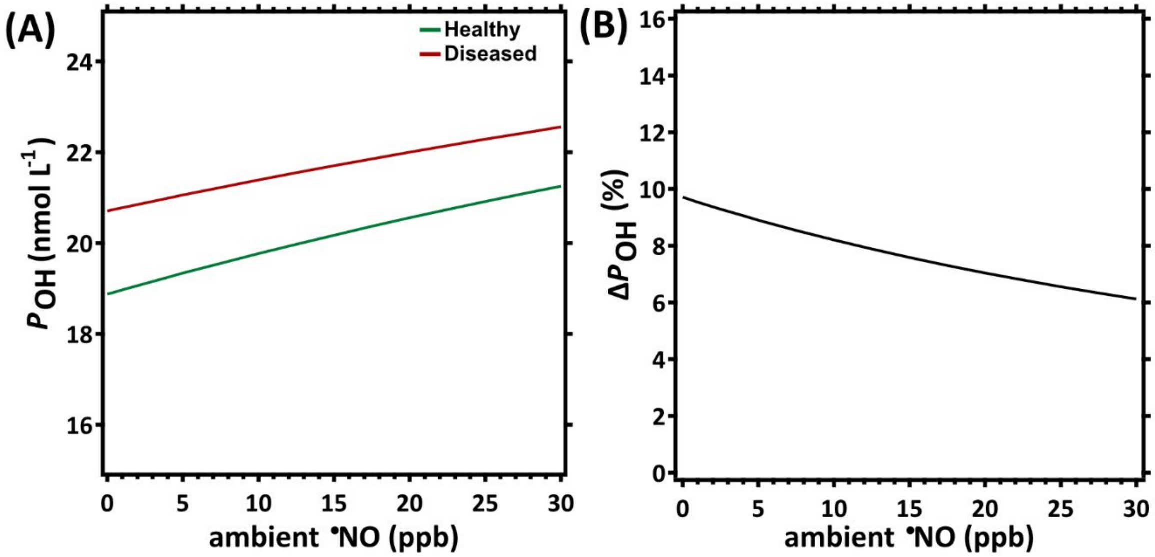

Figure S4: Sensitivity of P_{OH} on ambient •NO in healthy and diseased individuals with reference eNO concentrations of 10 ppb (green) and 30 ppb (red), respectively. We define reference eNO concentrations as the •NO mixing ratio exhaled at 0 ppb ambient •NO. Panel (B) shows the change in P_{OH} from a typical 3-fold increase in eNO between healthy and diseased individuals as a function of ambient •NO. Calculations are performed using the standard air pollution scenario for $PM_{2.5}$, NO_2 and O_3.

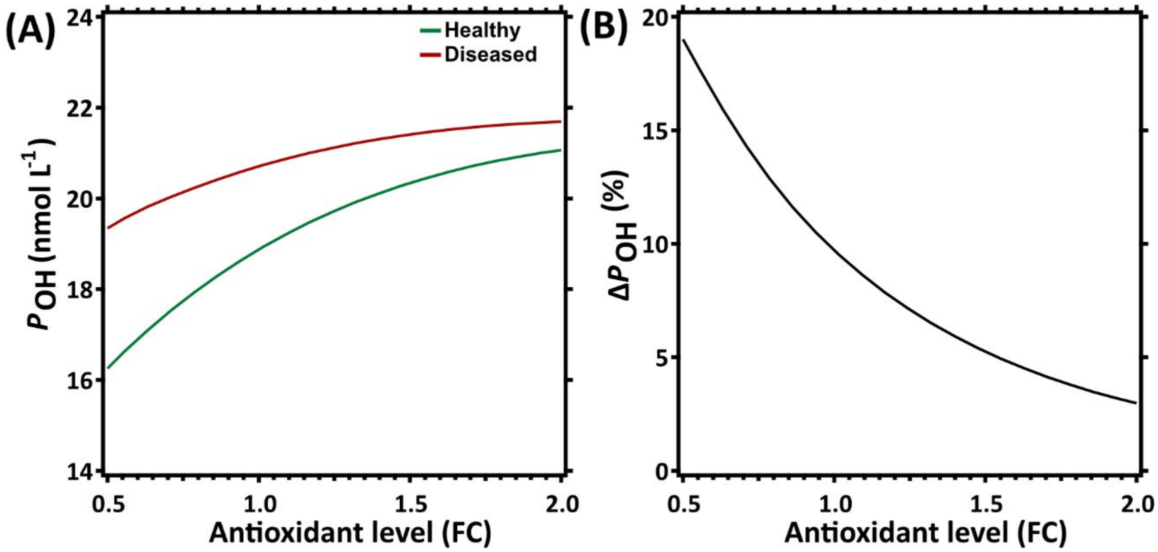

Figure S5: The effect of decreased and increased antioxidant levels on $^{\bullet}OH$ production, P_{OH}, in healthy and diseased individuals. All antioxidant concentrations are scaled with a factor (FC), including the low-molecular weight antioxidants ascorbate, glutathione and uric acid, as well as the enzymes catalase, SOD and GPx. For healthy and diseased individuals, we assume eNO concentrations of 10 and 30 ppb, respectively. Panel (B) shows the change in P_{OH} from a typical 3-fold increase in eNO between healthy and diseased individuals as a function of antioxidant levels. Calculations are performed using the standard air pollution scenario.

Table S1. Chemical reactions, rate constants as used in the KM-SUB-ELF, with reference.

#	Reaction	Rate constant (cm^{-3} s^{-1} or s^{-1})	Ref.
	Gas-phase reactions		
1	$NO^{\bullet} + O_3 \rightarrow NO_2^{\bullet} + O_2$	2.05×10^{-14}	[2,3]
2	$NO_2^{\bullet} + O_3 \rightarrow NO_3^{\bullet} + O_2$	4.85×10^{-17}	[2,3]
3	$NO^{\bullet} + NO^{\bullet} + O_2 \rightarrow NO_2^{\bullet} + NO_2^{\bullet}$	8.93×10^{-20}	[2,3]
4	$NO^{\bullet} + NO_3^{\bullet} \rightarrow NO_2^{\bullet} + NO_2^{\bullet}$	2.57×10^{-11}	[2,3]
5	$NO_2^{\bullet} + NO_3^{\bullet} \rightarrow NO^{\bullet} + NO_2^{\bullet} + O_2$	7.73×10^{-16}	[2,3]
6	$NO_2^{\bullet} + NO_3^{\bullet} \rightarrow N_2O_5$	1.21×10^{-12}	[2,3]
7	$^{\bullet}OH + O_3 \rightarrow HO_2^{\bullet} + O_2$	8.20×10^{-14}	[2,3]
8	$^{\bullet}OH + H_2O_2 \rightarrow HO_2^{\bullet} + H_2O$	1.73×10^{-12}	[2,3]
9	$HO_2^{\bullet} + O_3 \rightarrow {}^{\bullet}OH + O_2 + O_2$	8.24×10^{-16}	[2,3]
10	$^{\bullet}OH + HO_2^{\bullet} \rightarrow H_2O + O_2$	1.08×10^{-10}	[2,3]
11	$HO_2^{\bullet} + HO_2^{\bullet} \rightarrow H_2O_2 + O_2$	5.09×10^{-12}	[2,3]
12	$HO_2^{\bullet} + HO_2^{\bullet} \rightarrow H_2O_2$	3.50×10^{-12}	[2,3]
13	$^{\bullet}OH + NO^{\bullet} \rightarrow HONO$	8.91×10^{-12}	[2,3]

14	$^{\bullet}OH + NO_2^{\bullet} \rightarrow HNO_3$	8.91×10^{-12}	2,3
15	$^{\bullet}OH + NO_3^{\bullet} \rightarrow HO_2^{\bullet} + NO_2^{\bullet}$	2.00×10^{-11}	2,3
16	$HO_2^{\bullet} + NO^{\bullet} \rightarrow {}^{\bullet}OH + NO_2^{\bullet}$	8.24×10^{-12}	2,3
17	$HO_2^{\bullet} + NO_2^{\bullet} \rightarrow HO_2NO_2$	6.87×10^{-13}	2,3
18	$HO_2NO_2 \rightarrow HO_2^{\bullet} + NO_2^{\bullet}$	2.49×10^{-1}	2,3
19	$^{\bullet}OH + HO_2NO_2 \rightarrow NO_2^{\bullet} + H_2O + O_2$	2.96×10^{-12}	2,3
20	$HO_2^{\bullet} + NO_3^{\bullet} \rightarrow {}^{\bullet}OH + NO_2^{\bullet}$	4.00×10^{-12}	2,3
21	$^{\bullet}OH + HONO \rightarrow NO_2^{\bullet} + H_2O$	5.78×10^{-12}	2,3
22	$^{\bullet}OH + HNO_3 \rightarrow NO_3^{\bullet} + H_2O$	1.37×10^{-13}	2,3
23	$N_2O_5 \rightarrow NO_2^{\bullet} + NO_3^{\bullet}$	1.83×10^{-1}	2,3
	Surfactant reactions		
24	$SPB + {}^{\bullet}OH \rightarrow SPB\text{-}ox$	1.70×10^{-11}	21–23
25	$POG + {}^{\bullet}OH \rightarrow POG\text{-}ox$	1.70×10^{-11}	1
26	$SPB + O_3 \rightarrow SPB\text{-}ox$	1.00×10^{-14}	24,25
27	$POG + O_3 \rightarrow POG\text{-}ox + 0.17\ H_2O_2$	1.66×10^{-16}	26–29
28	$aToc + OH \rightarrow aToc\text{-}ox$	4.50×10^{-13}	30
29	$aToc + O_3 \rightarrow aToc\text{-}ox$	1.20×10^{-18}	31
	ELF reactions		
30	$O_2^{\bullet -} + HO_2 + H_2O \rightarrow H_2O_2 + OH^- + O_2$	1.70×10^{-13}	1,32
31	$HO_2 + HO_2 \rightarrow H_2O_2 + O_2$	1.40×10^{-15}	32
32	$O_2^{\bullet -} + O_2^{\bullet -} + 2H^+ \rightarrow H_2O_2 + O_2$	3.82×10^{-16}	32
33	$H_2O_2 + {}^{\bullet}OH \rightarrow HO_2 + H_2O$	5.50×10^{-14}	33
34	$^{\bullet}OH + {}^{\bullet}OH \rightarrow H_2O_2$	8.60×10^{-12}	34
35	$^{\bullet}OH + O_2^{\bullet -} \rightarrow O_2 + OH^-$	1.30×10^{-11}	21
36	$^{\bullet}OH + HO_2 \rightarrow H_2O + O_2$	1.20×10^{-11}	34
37	$H_2O_2 + HO_2 \rightarrow {}^{\bullet}OH + O_2 + H_2O$	4.98×10^{-21}	35
38	$Fe^{2+} + O_2^{\bullet -} + 2H^+ \rightarrow Fe^{3+} + H_2O_2$	3.10×10^{-14}	1,32
39	$Fe^{2+} + HO_2 + H^+ \rightarrow Fe^{3+} + H_2O_2$	1.99×10^{-15}	36
40	$Fe^{2+} + H_2O_2 \rightarrow Fe^{3+} + {}^{\bullet}OH + OH^-$	4.30×10^{-18}	37
41	$Fe^{2+} + {}^{\bullet}OH \rightarrow Fe^{3+} + OH^-$	5.30×10^{-13}	38
42	$Fe^{2+} + H_2O_2 \rightarrow Fe^{4+} + H_2O$	9.50×10^{-18}	1
43	$Fe^{3+} + H_2O_2 \rightarrow Fe^{2+} + HO_2 + H^+$	3.32×10^{-24}	37
44	$Fe^{3+} + HO_2 \rightarrow Fe^{2+} + O_2 + H^+$	3.30×10^{-18}	32
45	$Fe^{3+} + O_2^{\bullet -} \rightarrow Fe^{2+} + O_2 + H^+$	3.30×10^{-18}	32
46	$Fe^{4+} + Fe^{2+} \rightarrow Fe^{3+} + Fe^{3+}$	6.60×10^{-18}	39
47	$Fe^{3+} + AscH \rightarrow Fe^{2+} + Asc^{\bullet}$	1.10×10^{-19}	1
48	$Fe^{4+} + AscH \rightarrow Fe^{3+} + Asc^{\bullet}$	7.60×10^{-19}	1
49	$Fe^{2+} + O_2 \rightarrow O_2^{\bullet -} + Fe^{3+}$	5.20×10^{-21}	1
50	$Cu^+ + HO_2 + H^+ \rightarrow Cu^{2+} + H_2O_2$	2.30×10^{-12}	1
51	$Cu^+ + O_2^{\bullet -} + H_2O \rightarrow Cu^{2+} + H_2O_2 + OH^-$	5.80×10^{-15}	1
52	$Cu^{2+} + HO_2 \rightarrow Cu^+ + O_2 + H^+$	1.60×10^{-11}	1
53	$Cu^{2+} + O_2^{\bullet -} \rightarrow Cu^+ + O_2$	8.30×10^{-12}	1
54	$Cu^{2+} + AscH \rightarrow Cu^+ + Asc^{\bullet}$	1.40×10^{-18}	1

55	$Cu^+ + O_2 \rightarrow Cu^{2+} + O_2^{\bullet-}$	6.90×10^{-20}	1
56	$Cu^+ + H_2O_2 \rightarrow Cu^{2+} + {}^{\bullet}OH + OH^-$	2.40×10^{-20}	1
57	$Cu^+ + H_2O_2 \rightarrow Cu^{3+} + OH^- + OH^-$	5.00×10^{-19}	1
58	$Cu^+ + Cu^{3+} \rightarrow Cu^{2+} + Cu^{2+}$	5.80×10^{-12}	1
59	$Cu^{2+} + H_2O_2 \rightarrow Cu^+ + O_2^{\bullet-} + H^+$	3.80×10^{-24}	1
60	$PQN + AscH \rightarrow PQN^{\bullet} + Asc^{\bullet}$	1.20×10^{-20}	40
61	$PQN^{\bullet} + O_2 \rightarrow PQN + O_2^{\bullet-}$	4.60×10^{-13}	1
62	$PQN^{\bullet} + O_2^{\bullet-} + 2H^+ \rightarrow PQN + H_2O_2$	3.30×10^{-12}	1
63	$NQN12 + AscH \rightarrow NQN12^{\bullet} + Asc^{\bullet}$	1.50×10^{-19}	40
64	$NQN12^{\bullet} + O_2 \rightarrow NQN12 + O_2^{\bullet-}$	4.60×10^{-13}	1
65	$NQN12^{\bullet} + O_2^{\bullet-} + 2H^+ \rightarrow NQN12 + H_2O_2$	3.30×10^{-12}	1
66	$NQN14 + AscH \rightarrow NQN14^{\bullet} + Asc^{\bullet}$	6.30×10^{-21}	40
67	$NQN14^{\bullet} + O_2 \rightarrow NQN14 + O_2^{\bullet-}$	4.60×10^{-13}	1
68	$NQN14^{\bullet} + O_2^{\bullet-} + 2H^+ \rightarrow NQN14 + H_2O_2$	3.30×10^{-12}	1
69	$UA + O_3 \rightarrow$ Products	9.60×10^{-17}	31
70	$UA + {}^{\bullet}OH \rightarrow$ Products $+ OH^-$	1.20×10^{-11}	41
71	$GSH + {}^{\bullet}OH \rightarrow$ Products $+ OH^-$	1.50×10^{-11}	42
72	$GSSG + {}^{\bullet}OH \rightarrow$ Products $+ OH^-$	1.50×10^{-11}	8
73	$Asc^{\bullet} + Asc^{\bullet} + H^+ \rightarrow AscH + DHA$	5.00×10^{-16}	43
74	$AscH + O_2^{\bullet-} + H^+ \rightarrow Asc^{\bullet} + H_2O_2$	5.10×10^{-17}	1
75	$AscH + HO_2 \rightarrow Asc^{\bullet} + H_2O_2$	2.65×10^{-17}	44
76	$AscH + {}^{\bullet}OH \rightarrow$ Products $+ OH^-$	1.80×10^{-11}	45
77	$AscH + {}^{\bullet}O_3 \rightarrow$ Products	9.10×10^{-17}	31
78	$1.25\ GS^- + 0.5\ O_3 \rightarrow$ Products	9.60×10^{-20}	31
79	$1.25\ GSH + 0.5\ O_3 \rightarrow$ Products	9.60×10^{-20}	31
80	$GSOO^{\bullet} + GSOO^{\bullet} \rightarrow 0.56\ O_2^{\bullet-}$ + Products	6.79×10^{-13}	46
81	$O_2^{\bullet-} + GSH \rightarrow GSO^{\bullet} + OH^-$	3.32×10^{-19}	47–49
82	$NO_2^{\bullet} + GS^{\bullet} \rightarrow GSNO_2$	4.98×10^{-12}	50
83	$GSOO^{\bullet} + NO_2^{\bullet} \rightarrow GSOONO_2$	2.49×10^{-12}	46
84	$GSOONO_2 \rightarrow GSOO^{\bullet} + NO_2^{\bullet}$	7.5×10^{-1}	46
85	$NO_2^{\bullet} + GS^- \rightarrow NO_2^- + GS^{\bullet}$	4.00×10^{-13}	50
86	$NO_2^{\bullet} + GSH \rightarrow NO_2^- + GS^{\bullet} + H^+$	1.66×10^{-14}	51
87	$GSOO^{\bullet} + GSH \rightarrow GSO^{\bullet} + GSOH$	3.32×10^{-15}	50
88	$GSO + NO_2 \rightarrow GSOONO$	7.47×10^{-12}	50
89	$GSOONO \rightarrow$ Products	7.00×10^{2}	50
90	$GS^{\bullet} + GS^- \rightarrow GSSG^{\bullet-}$	1.59×10^{-14}	50,52
91	$GS^{\bullet} + O_2 \rightarrow GSOO^{\bullet}$	3.20×10^{-12}	52
92	$GSOO^{\bullet} \rightarrow GS^{\bullet} + O_2$	6.00×10^{5}	52
93	$GSSG^{\bullet-} \rightarrow GS^{\bullet} + GS^-$	1.60×10^{5}	50,52
94	$GSSG^{\bullet-} + O_2 \rightarrow GSSG + O_2^{\bullet-}$	8.30×10^{-12}	50,52
95	$GS^{\bullet} + GS^{\bullet} \rightarrow GSSG$	8.30×10^{-12}	52
96	$GSOH + GSH \rightarrow GSSG + H_2O$	1.20×10^{-18}	53
97	$GSO^{\bullet} + GSO^{\bullet} \rightarrow$ Products	9.96×10^{-14}	50

98	$GS^- + H_2O_2 \rightarrow GSOH + OH^-$	1.60×10^{-21}	53
99	$GS^\bullet + AscH \rightarrow GSH + Asc^\bullet$	1.00×10^{-12}	54,55
100	$UA + NO_2^\bullet \rightarrow UA^\bullet + NO_2^-$	3.00×10^{-14}	56,57
101	$AscH + NO_2^\bullet \rightarrow Asc^\bullet + NO_2^-$	5.80×10^{-14}	56,57
102	$UA^\bullet + AscH \rightarrow UA + Asc^\bullet$	1.70×10^{-15}	55
103	$GS^\bullet + UA \rightarrow GSH + UA^\bullet$	5.00×10^{-14}	51
104	$O_2^{\bullet -} + NO_2^\bullet \rightarrow O_2NOOm$	7.50×10^{-12}	50,58
105	$O_2NOO^- \rightarrow NO_2^- + O_2$	7.00×10^{-1}	50
106	$O_2NOO^- \rightarrow O_2^{\bullet -} + NO_2^\bullet$	1.10×10^{0}	50
107	$NO_2^\bullet + NO_2^\bullet \rightarrow N_2O_4$	7.50×10^{-13}	59
108	$N_2O_4 \rightarrow NO_2^\bullet + NO_2^\bullet$	6.90×10^{3}	59
109	$N_2O_4 + H_2O \rightarrow NO_2^- + NO_3^- + 2H^+$	1.00×10^{3}	50
110	$O_2^{\bullet -} + O_3 + H_2O \rightarrow {}^\bullet OH + 2O_2 + OH^-$	2.50×10^{-12}	60
111	$HO_2 + O_3 \rightarrow {}^\bullet OH + 2O_2$	1.66×10^{-17}	60
112	$NO_2^- + {}^\bullet OH \rightarrow NO_2^\bullet + OH^-$	8.80×10^{-12}	58
113	${}^\bullet OH + NO_2^\bullet \rightarrow NO_3^- + H^+$	7.50×10^{-12}	50
114	${}^\bullet OH + NO_2^\bullet \rightarrow ONOOH$	7.50×10^{-12}	50
115	$ONOOH \rightarrow NO_2^\bullet + {}^\bullet OH$	3.00×10^{-1}	50
116	$ONOOH \rightarrow NO_3^- + H^+$	7.00×10^{-1}	50
117	$ONOO^- + GSH \rightarrow NO_2^- + GSOH$	1.10×10^{-18}	61
118	$GSO^\bullet + NO_2^\bullet \rightarrow GSOONO$	7.50×10^{-12}	50
119	$GSOONO + H_2O \rightarrow$ Products	7.00×10^{2}	50
120	$ONOOH + AscH \rightarrow Im_1$	1.66×10^{-15}	62
121	$Im_1 \rightarrow ONOOH + AscH$	5.00×10^{2}	62
122	$Im_1 \rightarrow Im_2$	4.00×10^{1}	62
123	$Im_2 \rightarrow Im_1$	5.00×10^{0}	62
124	$Im_2 + AscH \rightarrow Asc + DHA + NO_2^- + H_2O$	1.66×10^{-19}	62
125	$Im_2 \rightarrow Asc + NO_3^- + H^+$	8.50×10^{-1}	62
126	$ONOOH + UA \rightarrow UA^{rad} + NO_2 +$ Products	2.60×10^{-19}	63
127	$O_2^{\bullet -} + SOD + H^+ \rightarrow 0.5\ H_2O_2 + SOD$	2.65×10^{-12}	8
128	$H_2O_2 +$ catalase $\rightarrow H_2O + 0.5\ O_2 +$ catalase	3.20×10^{-14}	8
129	$ONOOH +$ Glutathione peroxidase $\rightarrow NO_2^-$	1.33×10^{-14}	64
130	${}^\bullet OH +$ organic matter $\rightarrow$ oxidized organic matter	1.66×10^{-12}	41,65
131	$O_2^{\bullet -} + NO^\bullet \rightarrow ONOO^-$	3.16×10^{-11}	66
132	$ONOO^- + CO_2 \rightarrow ONOOCO_2^-$	4.98×10^{-17}	67
133	$ONOOCO_2^- \rightarrow NO_2^\bullet + CO_3^{\bullet -}$	3.3×10^{5}	50,67
134	$ONOOCO_2^- \rightarrow NO_3^- + CO_2$	6.7×10^{5}	50,67
135	$ONOO^- + GSH \rightarrow NO_2^- + GSOH$	2.24×10^{-18}	68
136	$NO_2^\bullet + NO^\bullet \rightarrow N_2O_3$	1.83×10^{-12}	67
137	$SOA \rightarrow {}^\bullet OH$	5.56×10^{-7}	*See SI text*
138	$SOA \rightarrow H_2O_2$	6.13×10^{-7}	*See SI text*
139	$SOA + Fe^{2+} \rightarrow {}^\bullet OH + Fe^{3+}$	7.90×10^{-23}	*See SI text*

Table S2. $PM_{2.5}$ and transition metal concentrations with mass fractions as quantified in $PM_{2.5}$ collected at different sampling sites throughout the world. Sampling sites used in Figure 4A are indicated with an asterisk and were selected to represent diverse parts of the globe, a large variability in $PM_{2.5}$ mass and composition.

**Sampling location**	**$PM_{2.5}$ (µg m^{-3})**	**Fe (ng m^{-3})**	**Cu (ng m^{-3})**	**Fe mass fraction**	**Cu mass fraction**	**Ref.**
Amazon (wet season)	1.65	33	0.07	2.00×10^{-2}	4.24×10^{-5}	[69]
Mace Head (Ireland)	4.2	5.68	0.71	1.35×10^{-3}	1.69×10^{-4}	[70]
Amazon (dry season)	4.87	19	0.8	3.90×10^{-3}	1.64×10^{-4}	[69]
*Edinburgh (Scotland)	7.1	27.6	1.39	3.89×10^{-3}	1.96×10^{-4}	[71]
West Midlands (UK, rural)	7.6	51.3	13.5	6.75×10^{-3}	1.78×10^{-4}	[72]
West Midlands (UK, urban)	9	80.2	13.9	8.91×10^{-3}	1.54×10^{-3}	[72]
Kruger National Park (South Africa)	9.4	51	0.41	5.43×10^{-3}	4.36×10^{-5}	[73]
Amazon (Serro do Navio)	9.87	120	1.65	1.22×10^{-2}	1.67×10^{-4}	[74]
Amazon (Cuiabá)	10.5	175	1.55	1.67×10^{-2}	1.48×10^{-4}	[74]
West Midlands (UK, rural, average)	10.5	87.1	20	8.30×10^{-3}	1.91×10^{-3}	[72]
West Midlands (UK, urban, average)	11.6	102	21.9	8.79×10^{-3}	1.89×10^{-3}	[72]
*Helsinki (Finland)	11.8	96	3.1	8.14×10^{-3}	2.63×10^{-4}	[75]
Tampa (Florida)	12.7	79	2.4	6.22×10^{-3}	1.89×10^{-4}	[76]
*Toronto (Canada)	12.7	55	2.5	4.33×10^{-3}	1.97×10^{-4}	[77]
South Phoenix (Texas)	12.95	147	7.6	1.14×10^{-2}	5.87×10^{-4}	[78]
Tehran (inside a school dormitory)	14	102.13	22.17	7.30×10^{-3}	1.58×10^{-3}	[79]
Tehran (inside a retirement home)	15	130.78	25.1	8.72×10^{-3}	1.67×10^{-3}	[79]
South-Eastern Italy (background sites)	16.4	86.8	3.1	5.29×10^{-3}	1.89×10^{-4}	[80]
*Patras (Greece)	17.4	124	7.28	7.13×10^{-3}	4.18×10^{-4}	[81]
Yeongwol (South Korea)	19.7	31.2	9.8	1.58×10^{-3}	4.98×10^{-4}	[82]
Budapest (Hungary)	20	430	18.3	2.15×10^{-2}	9.15×10^{-4}	[83]
South-Eastern Italy (industrial sites)	21.7	85	5.1	3.92×10^{-3}	2.35×10^{-4}	[80]
Zabrze (upper Silesia, Poland)	22	160.8	6.5	7.31×10^{-3}	2.96×10^{-4}	[84]
Chuncheon (South Korea)	23	29.6	9.9	1.29×10^{-3}	4.30×10^{-4}	[82]

*Detroit (Michigan)	23	234	6	1.02×10^{-2}	2.61×10^{-4}	85
Megalopolis (Greece)	23	87	4.02	3.78×10^{-3}	1.75×10^{-4}	81
Tehran (outside a retirement home)	24	238.81	25.99	9.95×10^{-3}	1.08×10^{-3}	79
South-Eastern Italy (urban sites)	24.1	78.8	5.7	3.27×10^{-3}	2.37×10^{-4}	80
Tehran (outside a school dormitory)	26	280	32.42	1.08×10^{-2}	1.25×10^{-3}	79
Anaheim (California)	26.8	29.6	39.6	1.11×10^{-3}	1.48×10^{-3}	86
Milan (Summer, Italy)	27.2	186	10	6.84×10^{-3}	3.68×10^{-4}	87
Jeddah City (Saudi Arabia)	28.4	590	5.6	2.08×10^{-2}	1.97×10^{-4}	88
*Hong Kong	29	140	5.7	4.83×10^{-3}	1.97×10^{-4}	89
*Rio de Janeiro (Brazil)	29.2	307	35	1.05×10^{-2}	1.20×10^{-3}	90
Katowice (upper Silesia, Poland)	31	157	8.2	5.07×10^{-3}	2.65×10^{-4}	84
Porto Marghera (Italy)	31	200	9.3	6.45×10^{-3}	3.00×10^{-4}	91
Erzgebirge (Germany)	32.5	188	3	5.79×10^{-3}	9.23×10^{-5}	92
*Barcelona (Spain)	35	260	52	7.43×10^{-3}	1.49×10^{-3}	93
Santa Catarina (Mexico)	36.15	466	16	1.29×10^{-2}	4.42×10^{-4}	94
Escobedo (Mexico)	37.78	493	13	1.31×10^{-2}	3.44×10^{-4}	94
Arnhem (the Netherlands)	38.95	241	13.5	6.19×10^{-3}	3.47×10^{-4}	95
Mira Loma (California, average)	41.8	581	75	1.39×10^{-2}	1.79×10^{-3}	96
Taif (residential area, Saudi Arabia)	46	2000	5.3	4.35×10^{-2}	1.15×10^{-4}	97
Taif (industrial site, Saudi Arabia)	47	2300	13	4.89×10^{-2}	2.77×10^{-4}	97
Azusa (California)	47.1	281.9	13.4	5.99×10^{-3}	2.85×10^{-4}	86
Edison (New Jersey)	49.6	1953	10	3.94×10^{-2}	2.02×10^{-4}	98
Bursa (Turkey)	53	875	15	1.65×10^{-2}	2.83×10^{-4}	99
Karachi (summer, Pakistan)	55.89	3360	56	6.01×10^{-2}	1.00×10^{-3}	100
*New Delhi (summer, India)	58.2	710	20	1.22×10^{-2}	3.44×10^{-4}	101
Milan (Winter, Italy)	58.6	309	18	5.27×10^{-3}	3.07×10^{-4}	87
Thessaloniki (cold period, Greece)	60.9	2890	93	4.75×10^{-2}	1.53×10^{-3}	102
Thessaloniki (warm period, Greece)	70.6	4094	66	5.60×10^{-2}	9.35×10^{-4}	102
Córdoba City (Argentina)	70.87	325	8	4.59×10^{-3}	1.13×10^{-4}	103
Shanghai (China)	71.61	424.93	9.47	5.93×10^{-3}	1.32×10^{-4}	104
Yong'an (Winter, China)	79.01	582.1	19.6	7.37×10^{-3}	2.48×10^{-4}	105

Yong'an (Spring, China)	83.26	736.1	16.5	8.84×10^{-3}	1.98×10^{-4}	[105]
Karachi (winter, Pakistan)	98.44	3706	39	3.77×10^{-2}	3.96×10^{-4}	[100]
Ji'nan (urban site, China)	101	1040	30	1.03×10^{-2}	2.97×10^{-4}	[106]
Guangzhou (China)	104.58	66	60	6.31×10^{-4}	5.74×10^{-4}	[107]
Argra (urban, India)	104.9	1900	200	1.81×10^{-2}	1.91×10^{-3}	[108]
Pune (India)	113.8	2090	339	1.84×10^{-2}	2.98×10^{-3}	[109]
Beijing (Summer, China)	125	1060	44.7	8.48×10^{-3}	3.58×10^{-4}	[110]
Ji'nan (industrial site, China)	130	2410	40	1.85×10^{-2}	3.08×10^{-4}	[106]
Beijing (Winter, China)	138	1330	53.2	9.64×10^{-3}	3.86×10^{-4}	[110]
*Beijing (China)	182.2	1180	70	6.48×10^{-3}	3.84×10^{-4}	[111]
New Delhi (winter, high traffic, India)	276.9	1150	70	4.15×10^{-3}	2.53×10^{-4}	[101]
*Dumai (peat fire episode, Indonesia)	640	4810	100	7.52×10^{-3}	1.56×10^{-4}	[112]
Iron and copper medians	–	–	–	8.14×10^{-3}	3.07×10^{-4}	[8]

Table S3. $PM_{2.5}$ and SOA concentrations with mass fractions as quantified in $PM_{2.5}$ collected at different sampling sites throughout the world.

Sampling location	$PM_{2.5}$ (μg m^{-3})	SOA (μg m^{-3})	SOA mass fraction	Ref.
Amazon (Brazil)	1.8	0.34	0.189	[113]
Hyytiälä (Finland)	2	1.2	0.600	[114]
Storm Peak (Colorado)	2.1	0.7	0.333	[114]
Jungfraujoch (Switzerland)	2.2	1.2	0.545	[114]
Duke Forest (North Carolina)	2.8	1.3	0.464	[114]
Chebogue Pt. (Canada)	2.9	1.5	0.517	[114]
Edinburgh (Scotland)	3	1.2	0.400	[114]
Mainz (Germany)	4.3	1.1	0.256	[114]
Boulder (Colorado)	4.4	2.5	0.568	[114]
Manchester (winter, UK)	5.2	0.6	0.115	[114]
Chelmsford (UK)	5.3	1.8	0.340	[114]
Vancouver (Canada)	7	2.5	0.357	[114]
Okinawa (Japan)	7.9	1.7	0.215	[114]
Off New England Coast	8.5	4.9	0.576	[114]
Thompson Farm (New Hampshire)	9.5	4.2	0.442	[114]
Zurich (winter, Switzerland)	9.6	4.3	0.448	[114]
Cheju (South Korea)	10.7	4	0.374	[114]
Fukue (Japan)	11	3.6	0.327	[114]
New York City (winter, New York)	11.6	2.6	0.224	[114]
New York City (summer, New York)	12.2	4.8	0.393	[114]
Pinnacle Park (New York)	12.3	5.4	0.439	[114]
Houston (Texas)	12.8	2.7	0.211	[114]
Tokyo (summer, Japan)	13.2	4.7	0.356	[114]
Manchester (summer, UK)	14.3	3	0.210	[114]
Pittsburgh (Pennsylvania)	14.7	3.1	0.211	[114]
Tokyo (winter, Japan)	16.2	2.3	0.142	[114]
Taunus (Germany)	16.3	7.9	0.485	[114]
Riverside (California)	19.1	7	0.366	[114]
Zurich (summer, Switzerland)	25.5	5.1	0.200	[114]
Mexico City (Mexico)	26.8	8.1	0.302	[114]
Guangzhou (China)	69.1	12.5	0.181	[115]
Beijing (China)	79.9	16.6	0.208	[115]
Shanghai (China)	90.7	11.1	0.122	[115]
Beijing (China)	158.5	40.9	0.258	[115]
Xi'an (China)	345.1	53.5	0.155	[115]
SOA median	–	–	*0.333*	[8]

Table S4. Phenanthrenequinone (PQN), 1,4-naphthoquinone (1,4-NQN) and 1,2-naphthoquinone (1,2-NQN) concentrations with mass fractions (MF) as quantified in $PM_{2.5}$ collected at different sampling sites throughout the world. Studies that did not report the $PM_{2.5}$ concentration at the sampling site are marked with an asterisk, and a concentration was estimated based on other observational data at that sampling site. Note, however, that these $PM_{2.5}$ concentrations are not used as input for calculations in this study.

Sampling location	$PM_{2.5}$ (µg m^{-3})	PQN (ng m^{-3})	1,2-NQN (ng m-3)	1,4-NQN (ng m^{-3})	PQN (MF)	1,2-NQN (MF)	1,4-NQN (MF)	Ref.
Umea (Sweden)	7.8	Unknown	Unknown	0.03	2.6×10^{-6}	1.3×10^{-6}	1.3×10^{-6}	[116]
Athens (Greece)	35.6	0.071	0.157	0.26	2.69×10^{-6}	5.95×10^{-6}	9.86×10^{-6}	[117]
Mazar-e Sharif (Afghanistan)	69	Unknown	Unknown	0.027	1.06×10^{-6}	5.28×10^{-7}	5.28×10^{-7}	[116]
Kabul (Afghanistan)	86	Unknown	Unknown	0.2	6.28×10^{-6}	3.14×10^{-6}	3.14×10^{-6}	[116]
Atascadero (California)	5*	0.023	0.0127	0.0246	6.21×10^{-6}	3.43×10^{-6}	6.64×10^{-6}	[118]
Birmingham (UK)	15*	4.6	3.2	1.7	4.14×10^{-4}	2.88×10^{-4}	1.53×10^{-4}	[119]
Lake Elsinore (California)	20*	0.311	0.246	0.14	2.1×10^{-5}	1.66×10^{-5}	9.45×10^{-6}	[118]
Norfolk (UK)	5*	0.058	0.024	0.012	1.57×10^{-5}	6.48×10^{-6}	3.24×10^{-6}	[120]
Riverside (California)	25*	0.57	0.06	0.23	3.08×10^{-5}	3.24×10^{-6}	1.24×10^{-5}	[121]
Quinones median	–	–	–	–	*6.28* $\times 10^{-6}$	*3.43* $\times 10^{-6}$	*6.64* $\times 10^{-6}$	[8]

References

(1) Lakey, P. S. J.; Berkemeier, T.; Tong, H.; Arangio, A. M.; Lucas, K.; Pöschl, U.; Shiraiwa, M. Chemical Exposure-Response Relationship between Air Pollutants and Reactive Oxygen Species in the Human Respiratory Tract. *Sci. Rep.* **2016**, *6* (1), 32916. https://doi.org/10.1038/srep32916.

(2) Saunders, S. M.; Jenkin, M. E.; Derwent, R. G.; Pilling, M. J. Protocol for the Development of the Master Chemical Mechanism, MCM v3 (Part A): Tropospheric Degradation of Non-Aromatic Volatile Organic Compounds. *Atmos. Chem. Phys.* **2003**, *3* (1), 161–180. https://doi.org/10.5194/acp-3-161-2003.

(3) Jenkin, M. E.; Saunders, S. M.; Wagner, V.; Pilling, M. J. Protocol for the Development of the Master Chemical Mechanism, MCM v3 (Part B): Tropospheric Degradation of Aromatic Volatile Organic Compounds. *Atmos. Chem. Phys.* **2003**, *3* (1), 181–193. https://doi.org/10.5194/acp-3-181-2003.

(4) Wang, C.; Liu, C.; Lin, H.; Yu, C.; Chung, K.; Kuo, H. Increased Exhaled Nitric Oxide in Active Pulmonary Tuberculosis Due to Inducible NO Synthase Upregulation in Alveolar Macrophages. *Eur. Respir. J.* **1998**, *11* (4), 809. https://doi.org/10.1183/09031936.98.11040809.

(5) Forstermann, U.; Sessa, W. C. Nitric Oxide Synthases: Regulation and Function. *Eur. Heart J.* **2012**, *33* (7), 829–837. https://doi.org/10.1093/eurheartj/ehr304.

(6) Pöschl, U.; Rudich, Y.; Ammann, M. Kinetic Model Framework for Aerosol and Cloud Surface Chemistry and Gas-Particle Interactions – Part 1: General Equations, Parameters, and Terminology. *Atmos. Chem. Phys.* **2007**, 35. https://doi.org/10.5194/acp-7-5989-2007.

(7) Kharitonov, S.; Alving, K.; Barnes, P. J. Exhaled and Nasal Nitric Oxide Measurements: Recommendations. *Eur. Respir. J.* **1997**, *10* (7), 1683–1693. https://doi.org/10.1183/09031936.97.10071683.

(8) Lelieveld, S.; Wilson, J.; Dovrou, E.; Mishra, A.; Lakey, P. S. J.; Shiraiwa, M.; Pöschl, U.; Berkemeier, T. Hydroxyl Radical Production by Air Pollutants in Epithelial Lining Fluid Governed by Interconversion and Scavenging of Reactive Oxygen Species. *Environ. Sci. Technol.* **2021**, *55* (20), 14069–14079. https://doi.org/10.1021/acs.est.1c03875.

(9) Wang, Y.; Kim, H.; Paulson, S. E. Hydrogen Peroxide Generation from α- and β-Pinene and Toluene Secondary Organic Aerosols. *Atmos. Environ.* **2011**, *45* (18), 3149–3156. https://doi.org/10.1016/j.atmosenv.2011.02.060.

(10) Tong, H.; Arangio, A. M.; Lakey, P. S. J.; Berkemeier, T.; Liu, F.; Kampf, C. J.; Brune, W. H.; Pöschl, U.; Shiraiwa, M. Hydroxyl Radicals from Secondary Organic Aerosol Decomposition in Water. *Atmos. Chem. Phys.* **2016**, *16* (3), 1761–1771. https://doi.org/10.5194/acp-16-1761-2016.

(11) Tong, H.; Lakey, P. S. J.; Arangio, A. M.; Socorro, J.; Kampf, C. J.; Berkemeier, T.; Brune, W. H.; Pöschl, U.; Shiraiwa, M. Reactive Oxygen Species Formed in Aqueous Mixtures of Secondary Organic Aerosols and Mineral Dust Influencing Cloud Chemistry and Public Health in the Anthropocene. *Faraday Discuss.* **2017**, *200*, 251–270. https://doi.org/10.1039/C7FD00023E.

(12) Mudway, I. S.; Kelly, F. J. Ozone and the Lung: A Sensitive Issue. *Mol. Asp. Med.* **2000**, *21* (1–2), 1–48. https://doi.org/10.1016/S0098-2997(00)00003-0.

(13) van der Vliet, A.; O'Neill, C. A.; Cross, C. E.; Koostra, J. M.; Volz, W. G.; Halliwell, B.; Louie, S. Determination of Low-Molecular-Mass Antioxidant Concentrations in Human

Respiratory Tract Lining Fluids. *Am. J. Physiol.* **1999**, *276* (2), L289–L296. https://doi.org/10.1152/ajplung.1999.276.2.L289.
(14) Dove, R. E.; Leong-Smith, P.; Roos-Engstrand, E.; Pourazar, J.; Shah, M.; Behndig, A. F.; Mudway, I. S.; Blomberg, A. Cigarette Smoke–Induced Induction of Antioxidant Enzyme Activities in Airway Leukocytes Is Absent in Active Smokers with COPD. *Eur. Clin. Respir. J.* **2015**, *2* (1), 27837. https://doi.org/10.3402/ecrj.v2.27837.
(15) Tunnicliffe, W.; Harrison, R.; Kelly, F.; Dunster, C.; Ayres, J. The Effect of Sulphurous Air Pollutant Exposures on Symptoms, Lung Function, Exhaled Nitric Oxide, and Nasal Epithelial Lining Fluid Antioxidant Concentrations in Normal and Asthmatic Adults. *Occ. Environ. Med.* **2003**, *60* (11), e15. https://doi.org/doi.org/10.1136/oem.60.11.e15.
(16) Kelly, F. J.; Tetley, T. D. Nitrogen Dioxide Depletes Uric Acid and Ascorbic Acid but Not Glutathione from Lung Lining Fluid. *Biochem. J.* **1997**, *325* (1), 95–99. https://doi.org/10.1042/bj3250095.
(17) Cantin, A. M.; Fells, G. A.; Hubbard, R. C.; Crystal, R. G. Antioxidant Macromolecules in the Epithelial Lining Fluid of the Normal Human Lower Respiratory Tract. *J. Clin. Invest.* **1990**, *86* (3), 962–971. https://doi.org/10.1172/JCI114798.
(18) Forman, H. J.; Fridovich, I. Superoxide Dismutase: A Comparison of Rate Constants. *Arch. Biochem. Biophys.* **1973**, *158* (1), 396–400. https://doi.org/10.1016/0003-9861(73)90636-X.
(19) Fridovich, I. Superoxide Dismutases. *Annu. Rev. Biochem.* **1975**, *44* (1), 147–159. https://doi.org/10.1146/annurev.bi.44.070175.001051.
(20) Comhair, S. A. A.; Lewis, M. J.; Bhathena, P. R.; Hammel, J. P.; Erzurum, S. C. Increased Glutathione and Glutathione Peroxidase in Lungs of Individuals with Chronic Beryllium Disease. *Am. J. Respir. Crit. Care Med.* **1999**, *159* (6), 1824–1829. https://doi.org/10.1164/ajrccm.159.6.9810044.
(21) Buxton, G. V.; Greenstock, C. L.; Helman, W. P.; Ross, A. B. Critical Review of Rate Constants for Reactions of Hydrated Electrons, Hydrogen Atoms and Hydroxyl Radicals ($\cdot OH/\cdot O^-$) in Aqueous Solution. *J. Phys. Chem. Ref. Data* **1988**, *17* (2), 513–886. https://doi.org/10.1063/1.555805.
(22) Hoffman, M. Z.; Hayon, E. Pulse Radiolysis Study of Sulfhydryl Compounds in Aqueous Solution. *J. Phys. Chem.* **1973**, *77* (8), 990–996. https://doi.org/10.1021/j100627a005.
(23) Zhao, M. J.; Jung, L.; Tanielian, C.; Mechin, R. Kinetics of the Competitive Degradation of Deoxyribose and Other Biomolecules by Hydroxyl Radicals Produced by the Fenton Reaction. *Free Rad. Res.* **1994**, *20* (6), 345–363. https://doi.org/10.3109/10715769409145635.
(24) Kanofsky, J. R.; Sima, P. D. Reactive Absorption of Ozone by Aqueous Biomolecule Solutions: Implications for the Role of Sulfhydryl Compounds as Targets for Ozone. *Arch. Biochem. Biophys.* **1995**, *316* (1), 52–62. https://doi.org/10.1006/abbi.1995.1009.
(25) Pryor, W. A.; Giamalva, D. H.; Church, D. F. Kinetics of Ozonation. 2. Amino Acids and Model Compounds in Water and Comparisons to Rates in Nonpolar Solvents. *J. Am. Chem. Soc.* **1984**, *106* (23), 7094–7100. https://doi.org/10.1021/ja00335a038.
(26) Kim, H. I.; Kim, H.; Shin, Y. S.; Beegle, L. W.; Jang, S. S.; Neidholdt, E. L.; Goddard, W. A.; Heath, J. R.; Kanik, I.; Beauchamp, J. L. Interfacial Reactions of Ozone with Surfactant Protein B in a Model Lung Surfactant System. *J. Am. Chem. Soc.* **2010**, *132* (7), 2254–2263. https://doi.org/10.1021/ja908477w.

(27) Hasson, A. S.; Ho, A. W.; Kuwata, K. T.; Paulson, S. E. Production of Stabilized Criegee Intermediates and Peroxides in the Gas Phase Ozonolysis of Alkenes: 2. Asymmetric and Biogenic Alkenes. *J. Geophys. Res.* **2001**, *106* (D24), 34143–34153. https://doi.org/10.1029/2001JD000598.

(28) Hewitt, C. N.; Kok, G. L. Formation and Occurrence of Organic Hydroperoxides in the Troposphere: Laboratory and Field Observations. *J. Atmos. Chem.* **1991**, *12* (2), 181–194. https://doi.org/10.1007/BF00115779.

(29) Zhou, Z.; Abbatt, J. P. D. Formation of Gas-Phase Hydrogen Peroxide via Multiphase Ozonolysis of Unsaturated Lipids. *Environ. Sci. Technol. Lett.* **2021**, *8* (2), 114–120. https://doi.org/10.1021/acs.estlett.0c00757.

(30) Navarrete, M.; Rangel, C.; Corchado, J. C.; Espinosa-García, J. Trapping of the OH Radical by α-Tocopherol: A Theoretical Study. *J. Phys. Chem. A* **2005**, *109* (21), 4777–4784. https://doi.org/10.1021/jp050717e.

(31) Kermani, S.; Ben-Jebria, A.; Ultman, J. S. Kinetics of Ozone Reaction with Uric Acid, Ascorbic Acid, and Glutathione at Physiologically Relevant Conditions. *Arch. Biochem. Biophys.* **2006**, *451* (1), 8–16. https://doi.org/10.1016/j.abb.2006.04.015.

(32) Rush, J. D.; Bielski, B. H. J. Pulse Radiolytic Studies of the Reaction of Perhydroxyl/Superoxide O2- with Iron(II)/Iron(III) Ions. The Reactivity of HO2/O2- with Ferric Ions and Its Implication on the Occurrence of the Haber-Weiss Reaction. *J. Phys. Chem.* **1985**, *89* (23), 5062–5066. https://doi.org/10.1021/j100269a035.

(33) Christensen, H.; Sehested, K.; Corfitzen, H. Reactions of Hydroxyl Radicals with Hydrogen Peroxide at Ambient and Elevated Temperatures. *J. Phys. Chem.* **1982**, *86* (9), 1588–1590. https://doi.org/10.1021/j100206a023.

(34) Sehested, K.; Rasmussen, O. L.; Fricke, H. Rate Constants of OH with HO2,O2-, and H2O2+ from Hydrogen Peroxide Formation in Pulse-Irradiated Oxygenated Water. *J. Phys. Chem.* **1968**, *72* (2), 626–631. https://doi.org/10.1021/j100848a040.

(35) Koppenol, W. H. The Haber-Weiss Cycle – 70 Years Later. *Redox Rep.* **2001**, *6* (4), 229–234. https://doi.org/10.1179/135100001101536373.

(36) Jayson, G. G.; Parsons, B. J.; Swallow, A. J. Oxidation of Ferrous Ions by Perhydroxyl Radicals. *J. Chem. Soc., Faraday Trans. 1* **1973**, *69*, 236–242. https://doi.org/10.1039/f19736900236.

(37) Lewis, S.; Lynch, A.; Bachas, L.; Hampson, S.; Ormsbee, L.; Bhattacharyya, D. Chelate-Modified Fenton Reaction for the Degradation of Trichloroethylene in Aqueous and Two-Phase Systems. *Environ.* **2009**, *26* (4), 849–859. https://doi.org/10.1089/ees.2008.0277.

(38) Stuglik, Z.; PawełZagórski, Z. Pulse Radiolysis of Neutral Iron(II) Solutions: Oxidation of Ferrous Ions by OH Radicals. *Radiation Physics and Chemistry (1977)* **1981**, *17* (4), 229–233. https://doi.org/10.1016/0146-5724(81)90336-8.

(39) Hug, S. J.; Leupin, O. Iron-Catalyzed Oxidation of Arsenic(III) by Oxygen and by Hydrogen Peroxide: PH-Dependent Formation of Oxidants in the Fenton Reaction. *Environ. Sci. Technol.* **2003**, *37* (12), 2734–2742. https://doi.org/10.1021/es026208x.

(40) Charrier, J. G.; McFall, A. S.; Richards-Henderson, N. K.; Anastasio, C. Hydrogen Peroxide Formation in a Surrogate Lung Fluid by Transition Metals and Quinones Present in Particulate Matter. *Environ. Sci. Technol.* **2014**, *48* (12), 7010–7017. https://doi.org/10.1021/es501011w.

(41) Masuda, T.; Shinohara, H.; Kondo, M. Reactions of Hydroxyl Radicals with Nucleic Acid Bases and the Related Compounds in Gamma-Irradiated Aqueous Solution. *J. Radiat.* **1978**, *16* (3), 153–161. https://doi.org/10.1269/jrr.16.153.
(42) Liphard, M.; Bothe, E.; Schulte-Frohlinde, D. The Influence of Glutathione on Single-Strand Breakage in Single-Stranded DNA Irradiated in Aqueous Solution in the Absence and Presence of Oxygen. *Int. J. Radiat. Biol.* **1990**, *58* (4), 589–602. https://doi.org/10.1080/09553009014551951.
(43) Carr, A.; Lykkesfeldt, J. *Vitamin C in Health and Disease*; MDPI-Multidisciplinary Digital Publishing Institute, 2018.
(44) Shen, J.; Griffiths, P. T.; Campbell, S. J.; Utinger, B.; Kalberer, M.; Paulson, S. E. Ascorbate Oxidation by Iron, Copper and Reactive Oxygen Species: Review, Model Development, and Derivation of Key Rate Constants. *Sci. Rep.* **2021**, *11* (1), 7417. https://doi.org/10.1038/s41598-021-86477-8.
(45) Adams, G. E.; Boag, J. W.; Currant, J.; Michael, B. D. *Absolute Rate Constants for the Reaction of the Hydroxyl Radical with Organic Compounds*; Pulse Radiolysis, 1965.
(46) Goldstein, S.; Lind, J.; Merenyi, G. Reaction of Organic Peroxyl Radicals with $^{\bullet}NO_2$ and $^{\bullet}NO$ in Aqueous Solution: Intermediacy of Organic Peroxynitrate and Peroxynitrite Species. *J. Phys. Chem. A* **2004**, *108* (10), 1719–1725. https://doi.org/10.1021/jp037431z.
(47) Jones, C. M.; Lawrence, A.; Wardman, P.; Burkitt, M. J. Electron Paramagnetic Resonance Spin Trapping Investigation into the Kinetics of Glutathione Oxidation by the Superoxide Radical: Re-Evaluation of the Rate Constant. *Free Radical Biology and Medicine* **2002**, *32* (10), 982–990. https://doi.org/10.1016/S0891-5849(02)00791-8.
(48) Winterbourn, C. C.; Metodiewa, D. The Reaction of Superoxide with Reduced Glutathione. *Archives of biochemistry and biophysics* **1994**, *314* (2), 284–290. https://doi.org/10.1006/abbi.1994.1444.
(49) Wefers, H.; Sies, H. Oxidation of Glutathione by the Superoxide Radical to the Disulfide and the Sulfonate Yielding Singlet Oxygen. *Eur J Biochem* **1983**, *137* (1–2), 29–36. https://doi.org/10.1111/j.1432-1033.1983.tb07791.x.
(50) Kirsch, M.; Lehnig, M.; Korth, H.-G.; Sustmann, R.; de Groot, H. Inhibition of Peroxynitrite-Induced Nitration of Tyrosine by Glutathione in the Presence of Carbon Dioxide through Both Radical Repair and Peroxynitrate Formation. *Chemistry–A European Journal* **2001**, *7* (15), 3313–3320. https://doi.org/10.1002/1521-3765(20010803)7:15<3313::aid-chem3313>3.0.co;2-7.
(51) Ford, E.; Hughes, M. N.; Wardman, P. Kinetics of the Reactions of Nitrogen Dioxide with Glutathione, Cysteine, and Uric Acid at Physiological PH. *Free Radical Biology and Medicine* **2002**, *32* (12), 1314–1323. https://doi.org/10.1016/S0891-5849(02)00850-X.
(52) Wardman, P.; Sonntag, C. [3] Kinetic factors that control the fate of thiyl radicals in cells. In *Methods in Enzymology*; Elsevier: gr, 1995; Vol. 251, pp 31–45. https://doi.org/10.1016/0076-6879(95)51108-3.
(53) Luo, D.; Smith, S. W.; Anderson, B. D. Kinetics and Mechanism of the Reaction of Cysteine and Hydrogen Peroxide in Aqueous Solution. *Journal of Pharmaceutical Sciences* **2005**, *94* (2), 304–316. https://doi.org/10.1002/jps.20253.
(54) Winkler, B. S.; Orselli, S. M.; Rex, T. S. The Redox Couple between Glutathione and Ascorbic Acid: A Chemical and Physiological Perspective. *Free Radical Biology and Medicine* **1994**, *17* (4), 333–349. https://doi.org/10.1016/0891-5849(94)90019-1.

(55) Buettner, G. R.; Jurkiewicz, B. A. Catalytic Metals, Ascorbate and Free Radicals: Combinations to Avoid. *Radiation Research* **1996**, *145* (5), 532. https://doi.org/10.2307/3579271.
(56) Alfassi, Z. B.; Huie, R. E.; Neta, P.; Shoute, L. C. T. Temperature Dependence of the Rate Constants for Reaction of Inorganic Radicals with Organic Reductants. *J. Phys. Chem.* **1990**, *94* (25), 8800–8805. https://doi.org/10.1021/j100388a011.
(57) Augusto, O.; Bonini, M. G.; Amanso, A. M.; Linares, E.; Santos, C. C. X.; De Menezes, S. L. Nitrogen Dioxide and Carbonate Radical Anion: Two Emerging Radicals in Biology. *Free Radical Biology and Medicine* **2002**, *32* (9), 841–859. https://doi.org/10.1016/S0891-5849(02)00786-4.
(58) Goldstein, S.; Czapski, G. Reactivity of Peroxynitrite versus Simultaneous Generation of $^{\bullet}NO$ and $O_2^{\bullet-}$ toward NADH. *Chem. Res. Toxicol.* **2000**, *13* (8), 736–741. https://doi.org/10.1021/tx000099n.
(59) Graetzel, M. Pulsradiolytische Untersuchung einiger Elementarprozesse der Oxydation und Reduktion des Nitritions. *Berichte der Bunsengesellschaft fuer Physikalische Chemie* **1969**, *73* (7), 646–653. https://doi.org/10.1002/bbpc.19690730707.
(60) Jacob, D. Heterogeneous Chemistry and Tropospheric Ozone. *Atmospheric Environment* **2000**, *34* (12–14), 2131–2159. https://doi.org/10.1016/S1352-2310(99)00462-8.
(61) Bonini, M. G.; Augusto, O. Carbon Dioxide Stimulates the Production of Thiyl, Sulfinyl, and Disulfide Radical Anion from Thiol Oxidation by Peroxynitrite. *J. Biol. Chem.* **2001**, *276* (13), 9749–9754. https://doi.org/10.1074/jbc.M008456200.
(62) Kurz, C. R.; Kissner, R.; Nauser, T.; Perrin, D.; Koppenol, W. H. Rapid Scavenging of Peroxynitrous Acid by Monohydroascorbate. *Free Radical Biology and Medicine* **2003**, *35* (12), 1529–1537. https://doi.org/10.1016/j.freeradbiomed.2003.08.012.
(63) Squadrito, G. L.; Cueto, R.; Splenser, A. E.; Valavanidis, A.; Zhang, H.; Uppu, R. M.; Pryor, W. A. Reaction of Uric Acid with Peroxynitrite and Implications for the Mechanism of Neuroprotection by Uric Acid. *Archives of Biochemistry and Biophysics* **2000**, *376* (2), 333–337. https://doi.org/10.1006/abbi.2000.1721.
(64) Briviba, K.; Kissner, R.; Koppenol, W. H.; Sies, H. Kinetic Study of the Reaction of Glutathione Peroxidase with Peroxynitrite. *Chem. Res. Toxicol.* **1998**, *11* (12), 1398–1401. https://doi.org/10.1021/tx980086y.
(65) Pryor, W. A. Oxy-Radicals and Related Species: Their Formation, Lifetimes, and Reactions. *Annual review of Physiology* **1986**, *48* (1), 657–667. https://doi.org/10.1146/annurev.ph.48.030186.003301.
(66) Nauser, T.; Koppenol, W. H. The Rate Constant of the Reaction of Superoxide with Nitrogen Monoxide: Approaching the Diffusion Limit. *J. Phys. Chem. A* **2002**, *106* (16), 4084–4086. https://doi.org/10.1021/jp025518z.
(67) Lancaster, J. R. Nitroxidative, Nitrosative, and Nitrative Stress: Kinetic Predictions of Reactive Nitrogen Species Chemistry Under Biological Conditions. *Chem. Res. Toxicol.* **2006**, *19* (9), 1160–1174. https://doi.org/10.1021/tx060061w.
(68) Radi, R. Oxygen Radicals, Nitric Oxide, and Peroxynitrite: Redox Pathways in Molecular Medicine. *Proc Natl Acad Sci USA* **2018**, *115* (23), 5839–5848. https://doi.org/10.1073/pnas.1804932115.
(69) Arana, A. A.; Artaxo, P.; Rizzo, L. V.; Bastos, W. Long Term Measurements of the Elemental Composition and Optical Properties of Aerosols in Amazonia. *E3S Web of Conferences* **2013**, *1*, 03005. https://doi.org/10.1051/e3sconf/20130103005.

(70) Birmili, W.; Allen, A. G.; Bary, F.; Harrison, R. M. Trace Metal Concentrations and Water Solubility in Size-Fractionated Atmospheric Particles and Influence of Road Traffic. *Environ. Sci. Technol.* **2006**, *40* (4), 1144–1153. https://doi.org/10.1021/es0486925.
(71) Heal, M. R.; Hibbs, L. R.; Agius, R. M.; Beverland, I. J. Total and Water-Soluble Trace Metal Content of Urban Background PM10, PM2.5 and Black Smoke in Edinburgh, UK. *Atmospheric Environment* **2005**, *39* (8), 1417–1430. https://doi.org/10.1016/j.atmosenv.2004.11.026.
(72) Harrison, R. M.; Yin, J. Chemical Speciation of PM2.5 Particles at Urban Background and Rural Sites in the UK Atmosphere. *J. Environ. Monit.* **2010**, *12* (7), 1404–1414. https://doi.org/10.1039/c000329h.
(73) Maenhaut, W.; Salma, I.; Cafmeyer, J.; Annegarn, H. J.; Andreae, M. O. Regional Atmospheric Aerosol Composition and Sources in the Eastern Transvaal, South Africa, and Impact of Biomass Burning. *J. Geophys. Res.* **1996**, *101* (D19), 23631–23650. https://doi.org/10.1029/95JD02930.
(74) Artaxo, P.; Gerab, F.; Yamasoe, M. A.; Martins, J. V. Fine Mode Aerosol Composition at Three Long-Term Atmospheric Monitoring Sites in the Amazon Basin. *J. Geophys. Res.* **1994**, *99* (D11), 22857–22868. https://doi.org/10.1029/94JD01023.
(75) Pakkanen, T. A.; Loukkola, K.; Korhonen, C. H.; Aurela, M.; Mäkelä, T.; Hillamo, R. E.; Aarnio, P.; Koskentalo, T.; Kousa, A.; Maenhaut, W. Sources and Chemical Composition of Atmospheric Fine and Coarse Particles in the Helsinki Area. *Atmospheric Environment* **2001**, *35* (32), 5381–5391. https://doi.org/10.1016/S1352-2310(01)00307-7.
(76) Olson, D. A.; Turlington, J.; Duvall, R. M.; McDow, S. R.; Stevens, C. D.; Williams, R. Indoor and Outdoor Concentrations of Organic and Inorganic Molecular Markers: Source Apportionment of PM2.5 Using Low-Volume Samples. *Atmospheric Environment* **2008**, *42* (8), 1742–1751. https://doi.org/10.1016/j.atmosenv.2007.11.035.
(77) Lee, P. K. H.; Brook, J. R.; Dabek-Zlotorzynska, E.; Mabury, S. A. Identification of the Major Sources Contributing to PM $_{2.5}$ Observed in Toronto. *Environ. Sci. Technol.* **2003**, *37* (21), 4831–4840. https://doi.org/10.1021/es026473i.
(78) Upadhyay, N.; Clements, A.; Fraser, M.; Herckes, P. Chemical Speciation of PM $_{2.5}$ and PM $_{10}$ in South Phoenix, AZ. *Journal of the Air & Waste Management Association* **2011**, *61* (3), 302–310. https://doi.org/10.3155/1047-3289.61.3.302.
(79) Hassanvand, M. S.; Naddafi, K.; Faridi, S.; Nabizadeh, R.; Sowlat, M. H.; Momeniha, F.; Gholampour, A.; Arhami, M.; Kashani, H.; Zare, A.; Niazi, S.; Rastkari, N.; Nazmara, S.; Ghani, M.; Yunesian, M. Characterization of PAHs and Metals in Indoor/Outdoor PM10/PM2.5/PM1 in a Retirement Home and a School Dormitory. *Science of The Total Environment* **2015**, *527–528*, 100–110. https://doi.org/10.1016/j.scitotenv.2015.05.001.
(80) Contini, D.; Cesari, D.; Donateo, A.; Chirizzi, D.; Belosi, F. Characterization of PM10 and PM2.5 and Their Metals Content in Different Typologies of Sites in South-Eastern Italy. *Atmosphere* **2014**, *5* (2), 435–453. https://doi.org/10.3390/atmos5020435.
(81) Manousakas, M.; Papaefthymiou, H.; Eleftheriadis, K.; Katsanou, K. Determination of Water-Soluble and Insoluble Elements in PM2.5 by ICP-MS. *Science of The Total Environment* **2014**, *493*, 694–700. https://doi.org/10.1016/j.scitotenv.2014.06.043.
(82) Han, Y.-J.; Kim, H.-W.; Cho, S.-H.; Kim, P.-R.; Kim, W.-J. Metallic Elements in PM2.5 in Different Functional Areas of Korea: Concentrations and Source Identification.

Atmospheric Research **2015**, *153*, 416–428. https://doi.org/10.1016/j.atmosres.2014.10.002.
(83) Maenhaut, W.; Raes, N.; Chi, X.; Cafmeyer, J.; Wang, W.; Salma, I. Chemical Composition and Mass Closure for Fine and Coarse Aerosols at a Kerbside in Budapest, Hungary, in Spring 2002. *X-Ray Spectrom.* **2005**, *34* (4), 290–296. https://doi.org/10.1002/xrs.820.
(84) Rogula-Kozłowska, W.; Błaszczak, B.; Szopa, S.; Klejnowski, K.; Sówka, I.; Zwoździak, A.; Jabłońska, M.; Mathews, B. PM2.5 in the Central Part of Upper Silesia, Poland: Concentrations, Elemental Composition, and Mobility of Components. *Environ Monit Assess* **2013**, *185* (1), 581–601. https://doi.org/10.1007/s10661-012-2577-1.
(85) Morishita, M.; Keeler, G. J.; Kamal, A. S.; Wagner, J. G.; Harkema, J. R.; Rohr, A. C. Identification of Ambient PM2.5 Sources and Analysis of Pollution Episodes in Detroit, Michigan Using Highly Time-Resolved Measurements. *Atmospheric Environment* **2011**, *45* (8), 1627–1637. https://doi.org/10.1016/j.atmosenv.2010.09.062.
(86) Chow, J. C.; Watson, J. G.; Fujita, E. M.; Lu, Z.; Lawson, D. R.; Ashbaugh, L. L. Temporal and Spatial Variations of PM2.5 and PM10 Aerosol in the Southern California Air Quality Study. *Atmospheric Environment* **1994**, *28* (12), 2061–2080. https://doi.org/10.1016/1352-2310(94)90474-X.
(87) Vecchi, R.; Marcazzan, G.; Valli, G.; Ceriani, M.; Antoniazzi, C. The Role of Atmospheric Dispersion in the Seasonal Variation of PM1 and PM2.5 Concentration and Composition in the Urban Area of Milan (Italy). *Atmospheric Environment* **2004**, *38* (27), 4437–4446. https://doi.org/10.1016/j.atmosenv.2004.05.029.
(88) Khodeir, M.; Shamy, M.; Alghamdi, M.; Zhong, M.; Sun, H.; Costa, M.; Chen, L.-C.; Maciejczyk, P. Source Apportionment and Elemental Composition of PM2.5 and PM10 in Jeddah City, Saudi Arabia. *Atmospheric Pollution Research* **2012**, *3* (3), 331–340. https://doi.org/10.5094/APR.2012.037.
(89) Hagler, G. S. W.; Bergin, M. H.; Salmon, L. G.; Yu, J. Z.; Wan, E. C. H.; Zheng, M.; Zeng, L. M.; Kiang, C. S.; Zhang, Y. H.; Schauer, J. J. Local and Regional Anthropogenic Influence on PM2.5 Elements in Hong Kong. *Atmospheric Environment* **2007**, *41* (28), 5994–6004. https://doi.org/10.1016/j.atmosenv.2007.03.012.
(90) Loyola, J.; Arbilla, G.; Quiterio, S. L.; Escaleira, V.; Minho, A. S. Trace Metals in the Urban Aerosols of Rio de Janeiro City. *J. Braz. Chem. Soc.* **2012**, *23* (4), 628–638. https://doi.org/10.1590/S0103-50532012000400007.
(91) Squizzato, S.; Masiol, M.; Visin, F.; Canal, A.; Rampazzo, G.; Pavoni, B. The PM2.5 Chemical Composition in an Industrial Zone Included in a Large Urban Settlement: Main Sources and Local Background. *Environ. Sci.: Processes Impacts* **2014**, *16* (8), 1913–1922. https://doi.org/10.1039/C4EM00111G.
(92) Matschullat, J.; Maenhaut, W.; Zimmermann, F.; Juliane Fiebig. Aerosol and Bulk Deposition Trends in the 1990's, Eastern Erzgebirge, Central Europe. *Atmospheric Environment* **2000**, *34* (19), 3213–3221. https://doi.org/10.1016/S1352-2310(99)00516-6.
(93) Querol, X.; Alastuey, A.; Rodriguez, S.; Plana, F.; Ruiz, C. R.; Cots, N.; Massagué, G.; Puig, O. PM10 and PM2.5 Source Apportionment in the Barcelona Metropolitan Area, Catalonia, Spain. *Atmospheric Environment* **2001**, *35* (36), 6407–6419. https://doi.org/10.1016/S1352-2310(01)00361-2.
(94) Martinez, M. A.; Caballero, P.; Carrillo, O.; Mendoza, A.; Mejia, G. M. Chemical Characterization and Factor Analysis of $PM_{2.5}$ in Two Sites of Monterrey, Mexico.

Journal of the Air & Waste Management Association **2012**, *62* (7), 817–827. https://doi.org/10.1080/10962247.2012.681421.
(95) Janssen, N. A. H.; Van Mansom, D. F. M.; Van Der Jagt, K.; Harssema, H.; Hoek, G. Mass Concentration and Elemental Composition of Airborne Particulate Matter at Street and Background Locations. *Atmospheric Environment* **1997**, *31* (8), 1185–1193. https://doi.org/10.1016/S1352-2310(96)00291-9.
(96) Na, K.; Cocker, D. R. Characterization and Source Identification of Trace Elements in PM2.5 from Mira Loma, Southern California. *Atmospheric Research* **2009**, *93* (4), 793–800. https://doi.org/10.1016/j.atmosres.2009.03.012.
(97) Shaltout, A. A.; Boman, J.; Al-Malawi, D. R.; Shehadeh, Z. F. Elemental Composition of PM2.5 Particles Sampled in Industrial and Residential Areas of Taif, Saudi Arabia. *Aerosol Air Qual. Res.* **2013**, *13* (4), 1356–1364. https://doi.org/10.4209/aaqr.2012.11.0320.
(98) Chow, J. C.; Watson, J. G.; Lu, Z.; Lowenthal, D. H.; Frazier, C. A.; Solomon, P. A.; Thuillier, R. H.; Magliano, K. Descriptive Analysis of PM2. 5 and PM10 at Regionally Representative Locations during SJVAQS/AUSPEX. *Atmospheric Environment* **1996**, *30* (12), 2079–2112. https://doi.org/10.1016/1352-2310(95)00402-5.
(99) Kendall, M.; Pala, K.; Ucakli, S.; Gucer, S. Airborne Particulate Matter (PM2.5 and PM10) and Associated Metals in Urban Turkey. *Air Qual Atmos Health* **2011**, *4* (3–4), 235–242. https://doi.org/10.1007/s11869-010-0129-9.
(100) Mansha, M.; Ghauri, B.; Rahman, S.; Amman, A. Characterization and Source Apportionment of Ambient Air Particulate Matter (PM2.5) in Karachi. *Science of The Total Environment* **2012**, *425*, 176–183. https://doi.org/10.1016/j.scitotenv.2011.10.056.
(101) Pant, P.; Shukla, A.; Kohl, S. D.; Chow, J. C.; Watson, J. G.; Harrison, R. M. Characterization of Ambient PM2.5 at a Pollution Hotspot in New Delhi, India and Inference of Sources. *Atmospheric Environment* **2015**, *109*, 178–189. https://doi.org/10.1016/j.atmosenv.2015.02.074.
(102) Tolis, E. I.; Saraga, D. E.; Filiou, K. F.; Tziavos, N. I.; Tsiaousis, C. P.; Dinas, A.; Bartzis, J. G. One-Year Intensive Characterization on PM2.5 Nearby Port Area of Thessaloniki, Greece. *Environ Sci Pollut Res* **2015**, *22* (9), 6812–6826. https://doi.org/10.1007/s11356-014-3883-7.
(103) López, M. L.; Ceppi, S.; Palancar, G. G.; Olcese, L. E.; Tirao, G.; Toselli, B. M. Elemental Concentration and Source Identification of PM10 and PM2.5 by SR-XRF in Córdoba City, Argentina. *Atmospheric Environment* **2011**, *45* (31), 5450–5457. https://doi.org/10.1016/j.atmosenv.2011.07.003.
(104) Cao, L.; Zeng, J.; Liu, K.; Bao, L.; Li, Y. Characterization and Cytotoxicity of PM<0.2, PM0.2–2.5 and PM2.5–10 around MSWI in Shanghai, China. *IJERPH* **2015**, *12* (5), 5076–5089. https://doi.org/10.3390/ijerph120505076.
(105) Yin, L.; Niu, Z.; Chen, X.; Chen, J.; Xu, L.; Zhang, F. Chemical Compositions of PM2.5 Aerosol during Haze Periods in the Mountainous City of Yong'an, China. *Journal of Environmental Sciences* **2012**, *24* (7), 1225–1233. https://doi.org/10.1016/S1001-0742(11)60940-6.
(106) Zhou, S.; Yuan, Q.; Li, W.; Lu, Y.; Zhang, Y.; Wang, W. Trace Metals in Atmospheric Fine Particles in One Industrial Urban City: Spatial Variations, Sources, and Health Implications. *Journal of Environmental Sciences* **2014**, *26* (1), 205–213. https://doi.org/10.1016/S1001-0742(13)60399-X.

(107) Wang, X.; Bi, X.; Sheng, G.; Fu, J. Chemical Composition and Sources of PM10 and PM2.5 Aerosols in Guangzhou, China. *Environ Monit Assess* **2006**, *119* (1–3), 425–439. https://doi.org/10.1007/s10661-005-9034-3.
(108) Kulshrestha, A.; Satsangi, P. G.; Masih, J.; Taneja, A. Metal Concentration of PM2.5 and PM10 Particles and Seasonal Variations in Urban and Rural Environment of Agra, India. *Science of The Total Environment* **2009**, *407* (24), 6196–6204. https://doi.org/10.1016/j.scitotenv.2009.08.050.
(109) Yadav, S.; Satsangi, P. G. Characterization of Particulate Matter and Its Related Metal Toxicity in an Urban Location in South West India. *Environ Monit Assess* **2013**, *185* (9), 7365–7379. https://doi.org/10.1007/s10661-013-3106-6.
(110) Song, S.; Wu, Y.; Jiang, J.; Yang, L.; Cheng, Y.; Hao, J. Chemical Characteristics of Size-Resolved PM2.5 at a Roadside Environment in Beijing, China. *Environmental Pollution* **2012**, *161*, 215–221. https://doi.org/10.1016/j.envpol.2011.10.014.
(111) Sun, Y.; Zhuang, G.; Wang, Y.; Han, L.; Guo, J.; Dan, M.; Zhang, W.; Wang, Z.; Hao, Z. The Air-Borne Particulate Pollution in Beijing—Concentration, Composition, Distribution and Sources. *Atmospheric Environment* **2004**, *38* (35), 5991–6004. https://doi.org/10.1016/j.atmosenv.2004.07.009.
(112) See, S. W.; Balasubramanian, R.; Rianawati, E.; Karthikeyan, S.; Streets, D. G. Characterization and Source Apportionment of Particulate Matter ≤ 2.5 Mm in Sumatra, Indonesia, during a Recent Peat Fire Episode. *Environ. Sci. Technol.* **2007**, *41* (10), 3488–3494. https://doi.org/10.1021/es061943k.
(113) Pöschl, U.; Martin, S. T.; Sinha, B.; Chen, Q.; Gunthe, S. S.; Huffman, J. A.; Borrmann, S.; Farmer, D. K.; Garland, R. M.; Helas, G.; Jimenez, J. L.; King, S. M.; Manzi, A.; Mikhailov, E.; Pauliquevis, T.; Petters, M. D.; Prenni, A. J.; Roldin, P.; Rose, D.; Schneider, J.; Su, H.; Zorn, S. R.; Artaxo, P.; Andreae, M. O. Rainforest Aerosols as Biogenic Nuclei of Clouds and Precipitation in the Amazon. *Science* **2010**, *329* (5998), 1513–1516. https://doi.org/10.1126/science.1191056.
(114) Jimenez, J. L.; Canagaratna, M. R.; Donahue, N. M.; Prevot, A. S. H.; Zhang, Q.; Kroll, J. H.; DeCarlo, P. F.; Allan, J. D.; Coe, H.; Ng, N. L.; Aiken, A. C.; Docherty, K. S.; Ulbrich, I. M.; Grieshop, A. P.; Robinson, A. L.; Duplissy, J.; Smith, J. D.; Wilson, K. R.; Lanz, V. A.; Hueglin, C.; Sun, Y. L.; Tian, J.; Laaksonen, A.; Raatikainen, T.; Rautiainen, J.; Vaattovaara, P.; Ehn, M.; Kulmala, M.; Tomlinson, J. M.; Collins, D. R.; Cubison, M. J.; E.; Dunlea, J.; Huffman, J. A.; Onasch, T. B.; Alfarra, M. R.; Williams, P. I.; Bower, K.; Kondo, Y.; Schneider, J.; Drewnick, F.; Borrmann, S.; Weimer, S.; Demerjian, K.; Salcedo, D.; Cottrell, L.; Griffin, R.; Takami, A.; Miyoshi, T.; Hatakeyama, S.; Shimono, A.; Sun, J. Y.; Zhang, Y. M.; Dzepina, K.; Kimmel, J. R.; Sueper, D.; Jayne, J. T.; Herndon, S. C.; Trimborn, A. M.; Williams, L. R.; Wood, E. C.; Middlebrook, A. M.; Kolb, C. E.; Baltensperger, U.; Worsnop, D. R. Evolution of Organic Aerosols in the Atmosphere. *Science* **2009**, *326* (5959), 1525–1529. https://doi.org/10.1126/science.1180353.
(115) Huang, R.-J.; Zhang, Y.; Bozzetti, C.; Ho, K.-F.; Cao, J.-J.; Han, Y.; Daellenbach, K. R.; Slowik, J. G.; Platt, S. M.; Canonaco, F.; Zotter, P.; Wolf, R.; Pieber, S. M.; Bruns, E. A.; Crippa, M.; Ciarelli, G.; Piazzalunga, A.; Schwikowski, M.; Abbaszade, G.; Schnelle-Kreis, J.; Zimmermann, R.; An, Z.; Szidat, S.; Baltensperger, U.; Haddad, I. E.; Prévôt, A. S. H. High Secondary Aerosol Contribution to Particulate Pollution during Haze Events in China. *Nature* **2014**, *514* (7521), 218–222. https://doi.org/10.1038/nature13774.

(116) Wingfors, H. Characterization of the Size-Distribution of Aerosols and Particle-Bound Content of Oxygenated PAHs, PAHs, and n-Alkanes in Urban Environments in Afghanistan. *Atmospheric Environment* **2011**, *45* (26), 4360–4369. https://doi.org/10.1016/j.atmosenv.2011.05.049.
(117) Valavanidis, A.; Fiotakis, K.; Vlahogianni, T.; Papadimitriou, V.; Pantikaki, V. Determination of Selective Quinones and Quinoid Radicals in Airborne Particulate Matter and Vehicular Exhaust Particles. *Environ. Chem.* **2006**, *3* (2), 118–123. https://doi.org/10.1071/EN05089.
(118) Eiguren-Fernandez, A.; Miguel, A. H.; Di Stefano, E.; Schmitz, D. A.; Cho, A. K.; Thurairatnam, S.; Avol, E. L.; Froines, J. R. Atmospheric Distribution of Gas- and Particle-Phase Quinones in Southern California. *Aerosol Science and Technology* **2008**, *42* (10), 854–861. https://doi.org/10.1080/02786820802339546.
(119) Delgado-Saborit, J. M.; Alam, M. S.; Godri Pollitt, K. J.; Stark, C.; Harrison, R. M. Analysis of Atmospheric Concentrations of Quinones and Polycyclic Aromatic Hydrocarbons in Vapour and Particulate Phases. *Atmospheric Environment* **2013**, *77*, 974–982. https://doi.org/10.1016/j.atmosenv.2013.05.080.
(120) Alam, M. S.; Delgado-Saborit, J. M.; Stark, C.; Harrison, R. M. Investigating PAH Relative Reactivity Using Congener Profiles, Quinone Measurements and Back Trajectories. *Atmos. Chem. Phys.* **2014**, *14* (5), 2467–2477. https://doi.org/10.5194/acp-14-2467-2014.
(121) Cho, A. K.; Di Stefano, E.; You, Y.; Rodriguez, C. E.; Schmitz, D. A.; Kumagai, Y.; Miguel, A. H.; Eiguren-Fernandez, A.; Kobayashi, T.; Avol, E.; Froines, J. R. Determination of Four Quinones in Diesel Exhaust Particles, SRM 1649a, and Atmospheric PM $_{2.5}$ Special Issue of *Aerosol Science and Technology* on Findings from the Fine Particulate Matter Supersites Program. *Aerosol Science and Technology* **2004**, *38* (sup1), 68–81. https://doi.org/10.1080/02786820390229471.
